# Vaccination against Foot-And-Mouth Disease: Do Initial Conditions Affect Its Benefit?

**DOI:** 10.1371/journal.pone.0077616

**Published:** 2013-10-04

**Authors:** Thibaud Porphyre, Harriet K. Auty, Michael J. Tildesley, George J. Gunn, Mark E. J. Woolhouse

**Affiliations:** 1 Epidemiology Group, Centre for Immunity, Infection and Evolution, University of Edinburgh, Ashworth Laboratories, Edinburgh, United Kingdom; 2 Epidemiology Research Unit, Scotland’s Rural College, Inverness, United Kingdom; 3 Centre for Complexity Science, Zeeman Building, University of Warwick, Coventry, United Kingdom; Inserm & Universite Pierre et Marie Curie, France

## Abstract

When facing incursion of a major livestock infectious disease, the decision to implement a vaccination programme is made at the national level. To make this decision, governments must consider whether the benefits of vaccination are sufficient to outweigh potential additional costs, including further trade restrictions that may be imposed due to the implementation of vaccination. However, little consensus exists on the factors triggering its implementation on the field. This work explores the effect of several triggers in the implementation of a reactive vaccination-to-live policy when facing epidemics of foot-and-mouth disease. In particular, we tested whether changes in the location of the incursion and the delay of implementation would affect the epidemiological benefit of such a policy in the context of Scotland. To reach this goal, we used a spatial, premises-based model that has been extensively used to investigate the effectiveness of mitigation procedures in Great Britain. The results show that the decision to vaccinate, or not, is not straightforward and strongly depends on the underlying local structure of the population-at-risk. With regards to disease incursion preparedness, simply identifying areas of highest population density may not capture all complexities that may influence the spread of disease as well as the benefit of implementing vaccination. However, if a decision to vaccinate is made, we show that delaying its implementation in the field may markedly reduce its benefit. This work provides guidelines to support policy makers in their decision to implement, or not, a vaccination-to-live policy when facing epidemics of infectious livestock disease.

## Introduction

Vaccination is an important weapon to fight against infectious disease, both in human and livestock populations. While social and behavioural constraints limit its implementation in humans [1,2], use of vaccination in livestock populations is predominantly driven by economic considerations. This is particularly true when disease is highly transmissible and can have major economic impact, such as foot and mouth disease (FMD), classical swine fever or highly pathogenic avian influenza. Indeed, in countries where these diseases are absent, epidemics are subject to strict control measures, dictated by both regional and national level policies. In this situation, the decision of whether to implement vaccination as part of control measures when facing incursion of such diseases is left at the national level. To make this decision, governments must consider whether the benefits of vaccination are sufficient to outweigh potential additional costs, including further trade restrictions that may be imposed due to the implementation of vaccination.

For FMD, prophylactic vaccination outside of an epidemic is not permitted within the EU, but reactive ring vaccination may be used alongside animal movement restrictions and “stamping out” measures (culling of infected premises (IPs) and farms that had epidemiological contact during the silent period and are potentially infected, known as dangerous contacts (DCs), which is required under EU legislation (Commission Regulation No. 2003/85/EC). When facing a FMD epidemic, vaccination may be implemented following two different strategies: firstly, as a measure to restrict disease spread as quickly as possible, followed by culling of all vaccinated animals. This approach, known as “vaccination-to-cull” or suppressive vaccination, was used in Netherlands to control FMD in 2001 [3]. The second option is known as “vaccination-to-live” or protective vaccination, and does not require culling of vaccinated animals. This approach is widely cited by policy makers as part of future FMD control strategies, but it has not yet been used in EU. Field data to assess the impact of vaccination on controlling the outbreak are therefore not available, and decision making on vaccination strategies relies on computer simulations to assess its likely benefits.

Despite the obvious logistical and social advantages the vaccination may conceive by reducing the number of animals culled due to disease control purposes, there are inherent factors which may offset the likely effectiveness of a vaccination strategy. For example, the vaccine requires 4-5 days for immunity to develop and the vaccine efficacy is related to the antigenic match between the vaccine strain and the circulating strain [4]. These limitations render the effectiveness of vaccination policies very sensitive to the initial conditions of an epidemic [5–8]. Complementing the required control measures with vaccination-to-live has the potential to reduce disease spread by removing the susceptible population, leading to a smaller number of IPs, shorter epidemics, and fewer animals culled. However, models have suggested that the benefit of vaccination varies with the specific conditions of the outbreak [7,8]. The reasons for this variation have not been fully characterised and it remains unclear whether real benefit can be obtained when applying vaccination in different incursion scenarios. This makes it difficult for policy-makers to make definitive decisions regarding whether vaccination should be implemented [9].

In this paper, we use a modelling framework to assess how robust the benefits of vaccination are to specific outbreak conditions. In particular we investigate how the location of the incursion and the timing of its field implementation alter the benefit of a vaccination policy against an infectious disease such as FMD. To reach this goal, we based our analysis in the context of Scotland and used a spatial, premises-based model that has been extensively used to investigate the effectiveness of mitigation procedures in Great Britain [6,10].

## Methods

### Modelling framework

We used the fully stochastic, spatial, premises-based model that was developed and used during the FMD epidemic in 2001 in Great Britain [6,10]. This model has been extensively used to investigate the value of specific culling and vaccination strategies with respect to variations in epidemics conditions and control responses [6,11–13]. Hence, we refer to [6,10,11,14] for further details on the model.

**Figure 1:**
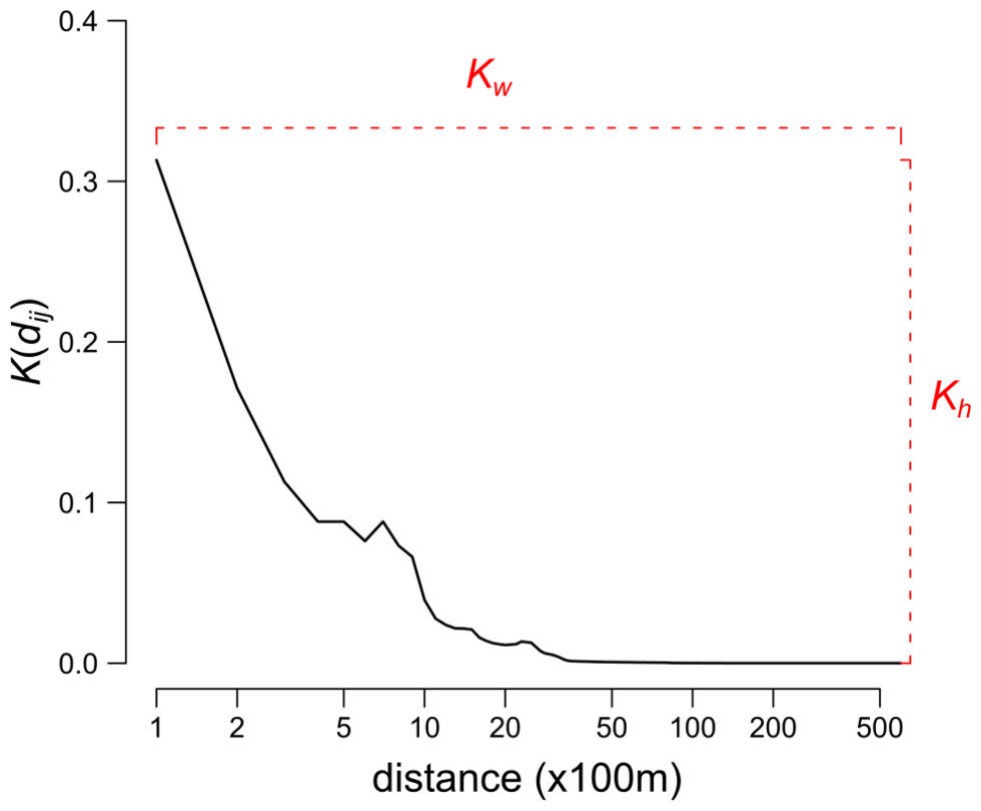
Between-farm transmission kernel function. Probability at which a given infected farm may infect a susceptible farm as a function of inter-farm distance rate *d*_*ij*_. Note that the x-axis is log scaled. Are also indicated the two main features that were used in the report to describe the shape of the transmission kernel function: the kernel’s height *K*_*h*_ and width *K*_*w*_.

Briefly, premises pass through four epidemiological states; they are either: susceptible; infected but not infectious; infectious; or reported infected and thereby culled. The model assumes that each *i* ^th^ premise would be infected with a daily probability depending on its own susceptibility *S*_*i*_ and on the transmissibility *T*_*j*_ of the surrounding *j* premises. For the *n* premises involved in the study population, each *i* ^th^ premise has a daily probability*M*_*i*_ to be infected such as


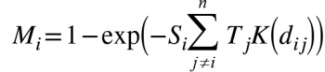
(1)

where *S*_*i*_ and *T*_*j*_ depend on the species (i.e. cattle and sheep) and on related herd size present on premise. The component *K*(*d_ij_*) of equation (1) denotes the “between-farm transmission kernel function” and determines the scaling factor on the rate at which an infected premise may infect susceptible ones as a function of inter-farm distance *d*_*ij*_. Two main features can be extracted from the shape of *K*(*d_ij_*): the kernel’s height *K*_*h*_ and width *K*_*w*_ (see Figure 1 for details). These two features inform the between-herd behaviour of the infection; indicating whether neighbouring premises are more likely to be infected (*K_h_*) or whether the infection may spread over a large distance (*K_w_*).

Both the susceptibility *S*_*i*_ of a given premise *i*, and the transmissibility *T*_*j*_ of those that are surrounding it, are computed such as:

$$S_{i}=s_{cow}N_{cow,i}^{p_{c}}+s_{sheep}N_{sheep,i}^{p_{s}}$$ (2)

$$T_{j}=t_{cow}N_{cow,j}^{q_{c}}+t_{sheep}N_{sheep,j}^{q_{s}}$$ (3)

The parameters of *s* and *t* in equations (2) and (3) correspond to the susceptibility and transmissibility of a farm per head of livestock recorded present on premise during the study period. Herd size and structure are given by the parameters *N*_*cow,i*_ and *N*_*sheep,i*_ for each premise *i*. In concordance with [14,15], but in contrast with the earlier implementation of the model [6,10,11], we used the power law parameters *p*_*s*_, *p*_*c*_, *q*_*s*_ and *q*_*c*_ to account for the nonlinear dependence of animal numbers upon susceptibility and transmissibility of a farm.

In the UK version of the model, the seven parameters *s*_*cow*_, *t*_*cow*_, *t*_*sheep*_, *p*_*s*_, *p*_*c*_, *q*_*s*_ and *q*_*c*_ (with *s*_*sheep*_ fixed to 1) were determined for five distinct regions (Cumbria, Devon, Scotland, Wales and the rest of GB) by fitting the model to the UK 2001 epidemics. In our study, all parameters involved in the model are therefore the Scotland-specific parameters (*s*_*cow*_ = 10.771, *s*_*sheep*_ = 1, *t*_*cow*_ = 8.37e-07, *t*_*sheep*_ *= 9.69e-07*, *p*_*s*_ = 0.326, *p*_*c*_ = 0.227, *q*_*s*_ = 0.403 and *q*_*c*_ = 0.202) as defined by Tildesley’s work [14,15]. We further assume that the spatial extent of the transmissibility between farms in Scotland is similar to that recorded during the 2001 UK FMD epidemic. Therefore, as a baseline, we used the shape of the transmission kernel function that was empirically derived from the contact tracing performed by DEFRA during the 2001 UK FMD epidemic once movement restrictions were implemented.

In line with previous versions of the model [6,10,11], we make the assumption that all farms are infected for 5 days before becoming infectious, and are infectious for 4 days before being reported with infection. As a baseline, the model considers that once an infected premise (IP) is reported, a national movement ban (NMB) would be enforced and culling measures would be implemented within 24 hours. In addition to the routine culling of IPs, premises where animals have been in direct contact with infected animals or have, in any way, become exposed to infection, known as dangerous contacts (DCs), are culled in an effort to control disease. Premises defined as DCs are determined based upon both prior infection by an IP and future risk of infection in the same way as in [6]. All farms defined as DCs in our model would be depopulated within 48 hours. Once animals at an IP are slaughtered, disinfection procedures are initiated and no transmission events to other premises may occur. For the purpose of this work, pre-emptive culling based on spatial proximity (also known as “contiguous premise” culling) was not considered.

### Farm data

Here we restrict our analysis of the efficiency of a vaccinate-to-live strategy only from a Scottish perspective. The model therefore takes into account only farms that are present within the boundaries of Scotland as informed by the Scottish Agricultural Census June 2011. Furthermore, the risk of infection between infectious and susceptible premises is drastically reduced if major geographical features intervene [16]. Because of the existence of natural barriers, together with the increased rigour of the implementation of biosecurity measures in harbours and airports during a FMD epidemic, we considered that premises located in the Scottish Islands would have little involvement in the spread of FMD and they were therefore discarded from the model. The extent of our study area and the distribution of premises and livestock that are recorded in the Scottish Agricultural Census June 2011 and considered in this exercise are shown in Table S1.

In 2001, there was little involvement of pig farms in the disease transmission dynamics, despite initiating the epidemic [17]. Consequently, whilst acknowledging that some strains of FMD can rapidly spread within pig populations [18,19], we assumed that the spread of FMD between pig farms in Scotland would be limited. This assumption is based on the relative resistance of pigs to infection by aerosols [20–22], the strict biosecurity generally carried out on commercial pig farms, and legislation unrelated to FMD that regulates the movement of swine from farm to farm [17]. We further considered that the high awareness of FMD amongst Scottish pig farmers would increase the likelihood of rapid detection should a pig farm become infected. We therefore considered that the FMD virus strain would only circulate within the cattle and sheep industry, though pig premises may still be subject to slaughter for disease control purposes.

### Ring vaccination and vaccination benefit

In line with the Scottish Government FMD contingency plan, we assumed that only cattle would be vaccinated against FMD [23] and vaccinated animals would become totally immune to infection after four days. As in previous work [6], we make the conservative assumption that during this four-day delay, all cattle are completely susceptible and if infected, the disease progresses in the same way as for non-vaccinated cattle. Unless otherwise stated, we considered that 90% of cattle present on vaccinated farms would become totally immune, while the rest would remain totally susceptible to be infected and transmit the virus. In other words, premises in which 90% of the cattle are immune were considered to have the same transmission and susceptibility properties as those with 10% of the number of cattle [6]. In addition, latent and infectious periods of vaccinated infected premises remain similar to those that were not vaccinated (i.e. 5 and 4 days, respectively).

Following the Scottish Government contingency plan, if the decision to vaccinate livestock is made, the model assumes a fixed 10km ring vaccination would be implemented around each IP [23]. Five days are required for vaccination teams to be mobilised in the fields. Unless otherwise stated, vaccination therefore commences seven days after the disease is first detected. Once vaccination teams are mobilised and actively deployed in the field, ring-vaccination around detected IPs would however be carried out within the recommended 24 hours [8].

We considered that premises were vaccinated as follows: (1) vaccination rings were tackled in the order of reporting of the associated IPs; and (2) for those farms within each vaccination ring, based on their distance from the IP, with priority given to the furthest. In other words, vaccination within each ring is performed from the outside in. Such a strategy corresponds to the standard policy but was shown differing little from an inside-out strategy in regards to the associated epidemic impact [6]. The model also assumes that a decision to vaccinate will be maintained throughout the outbreak and as disease spreads to new areas new vaccination zones will be created.

We assumed that a fixed number of vaccination teams would be mobilised to respond to an emergency (*n*=50, in line with current contractual arrangements that the Scottish Government have made in the event of an outbreak); each of which can vaccinate up to 250 animals per day [24]. This corresponds to a maximum of 12,500 animals vaccinated per day, which would limit the implementation of the vaccination strategy to 136 farms per day (on average). We further considered that the number of doses available to Scotland is the same as what is available for the whole of GB.

For each parameter set *k*, the ‘benefit of the vaccination-to-live strategy’ (VB) was computed in relative terms by comparing the epidemic impact when vaccination is implemented in complement to routine mitigation procedures (i.e. NMB + IP/DC culling), *c*_*v*_, with the epidemic impact when it is not, *c*_0_, such as:

$${\text{VB}}_{k}=1-\frac{{\hat{c}}_{v,k}}{{\hat{c}}_{0,k}}$$ (4) ,

where ${\hat{c}}_{v}$ and ${\hat{c}}_{0}$are the geometric means of the epidemic impact when vaccination is implemented in complement to routine mitigation procedures and when routine mitigation procedures are implemented alone, respectively. The value of VB can therefore be interpreted as the proportion of the epidemic impact that was saved due to the implementation of a vaccination policy.

In this work, several definitions of epidemic impact are used; we define the impact of an epidemic either by its duration (in days), by the number of premises that were infected and culled, by the total number of animals (cattle, sheep or pigs) that have been culled due to control activities or by the total number of cattle only that have been culled due to control activities. Furthermore, it is worth noting that we used the geometric mean of the each generated distribution to summarise the epidemic impact and, thus, inform on the average dynamic of the tested epidemiologic processes. The geometric mean was used rather than arithmetic mean to account for the considered distributions being mostly log-normal.

### Control scenarios

To respond to our objectives, two different scenarios were explored to evaluate variations of VB in terms of the key epidemiological uncertainties when facing a FMD epidemic in Scotland. First, we investigated the impact of varying the location of the initial incursion on VB. As such, epidemics were started by randomly infecting a single premise in each of the 31 studied Scottish counties in turn. For each county, 10,000 epidemics were simulated, allowing each of them to spread within the whole country. In the case where a decision to use vaccination is made, vaccination would be implemented in the field 7 days after the first reported case in addition to the implementation of routine control measures (i.e. NMB + IP/DC culling). Secondly, we focused on the counties that illustrated best the different epidemiological areas identified in the previous scenario to investigate the role of field implementation delay on VB. Again, epidemics were initiated by randomly infecting one premise. For each day increase in delay in implementing vaccination, 10,000 epidemics were then simulated. It is important to note that that while incursion events are located in a given (unique) county, all herds present in Scotland would be susceptible to infection.

### Sensitivity analyses

We assessed the sensitivity of VB with respect to variations in (1) quality of disease surveillance and (2) FMD virus characteristics. To test the resilience of the vaccination policy to changes in surveillance intensity, simulations were initialised with an increasing number of premises that are infected, to mimic late detection. In each situation, initially infected premises are randomly selected within the same county, and their infection time randomly allocated. Routine mitigation procedures are then activated once the first infected premise is detected and reported, while the remaining initial IPs would remain silently infected until disclosure. To investigate the resilience of the vaccination policy to changes in FMD virus characteristics, we explored the effect of varying the vaccine efficacy, the susceptibility and infectiousness parameters for cattle, and the height (*K*_*h*_) and width (*K*_*w*_) of the dispersal kernel function, generating 1000 epidemics for each considered combination of parameters. Varying the susceptibility and infectiousness parameters as well as the shape of the transmission kernel function explores the changes in the total transmission rate of the virus, its species specificity (cattle vs. sheep) and the distance over which it could be transmitted. In contrast, varying the vaccine efficacy explores the resilience of the vaccinate-to-live strategy to inherent limitations of the available vaccine due to imperfect FMD virus antigenic match between the vaccine strain and the circulating strain.

## Results

### Epidemiologic impact

For all incursion (*n*=310,000) involving a rapid detection of incursion events, infection seldom spread after the initial incursion in Scotland. When looking at epidemics arising from an initial case in each county of Scotland, on average, an epidemic would last for approximately 11 days with less than 3 infected premises (2.87) and less than 1500 animals culled (1168), including about 310 cattle, for disease control purposes. However, there are very large variations in the size and duration, with the most severe epidemics reaching a maximum of 1109 infected premises or lasting for more than 630 days. Although epidemics of more than 1000 IPs were extremely rare (less than once in 3000 incursions), the consequences would be disastrous for the Scottish livestock industry, with between 1.9 and 2.5 million animals culled for disease control purposes, representing 24% to 31% of the Scottish national herd. Overall, 8.8% and 11.3% of the generated outbreaks would result in epidemics of more than 100 infected premises or lasting more than 60 days, respectively.

### Spatial variation in benefit

**Figure 2:**
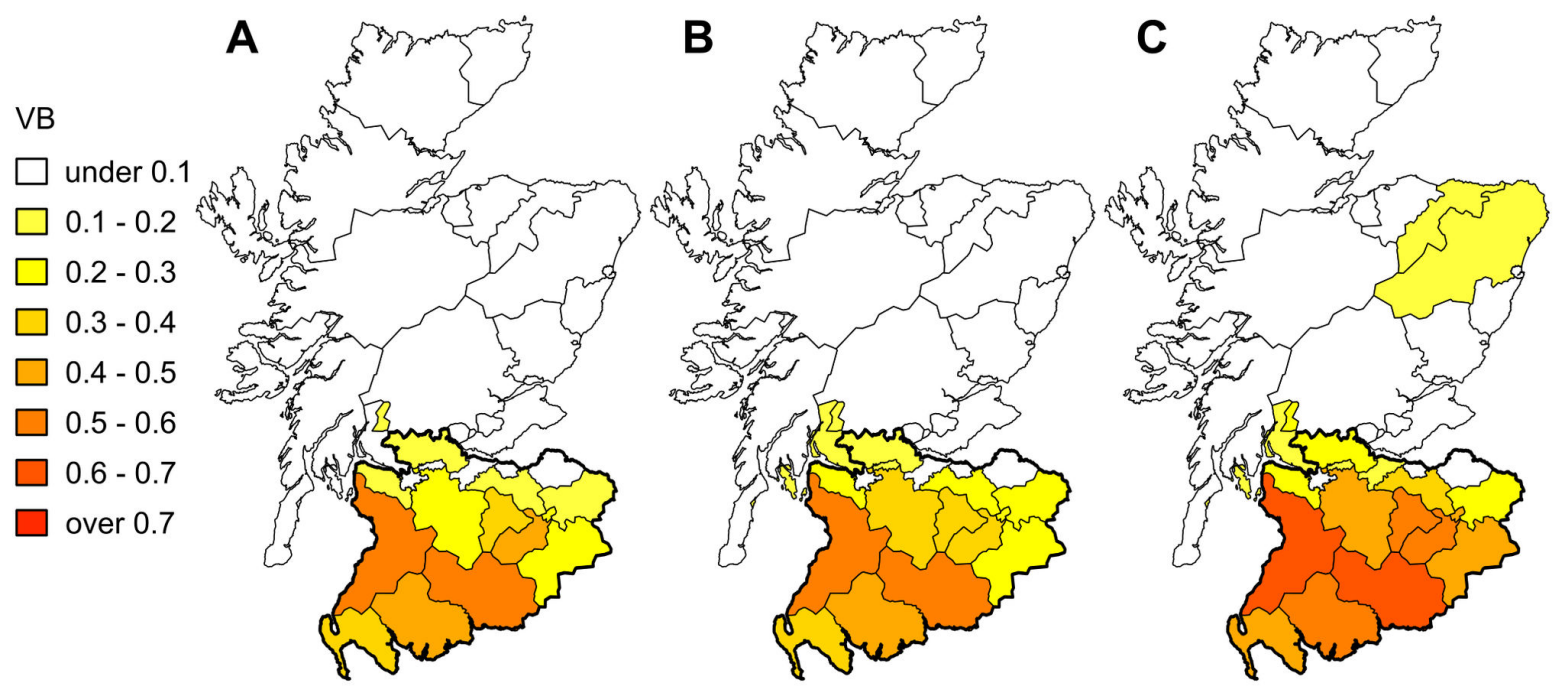
Spatial pattern in the benefit of the vaccination (VB) policy. Proportions of infected premises (A), animals culled (B) and cattle culled (C) that would be saved by implementing the vaccination policy in the field at 7 days in comparison with a strategy involving the culling of IP/DC premises alone. The darker the colour, the more vaccination is beneficial. Thick contour indicates counties defined as “southern counties”, which showed high epidemic impact.

**Figure 3:**
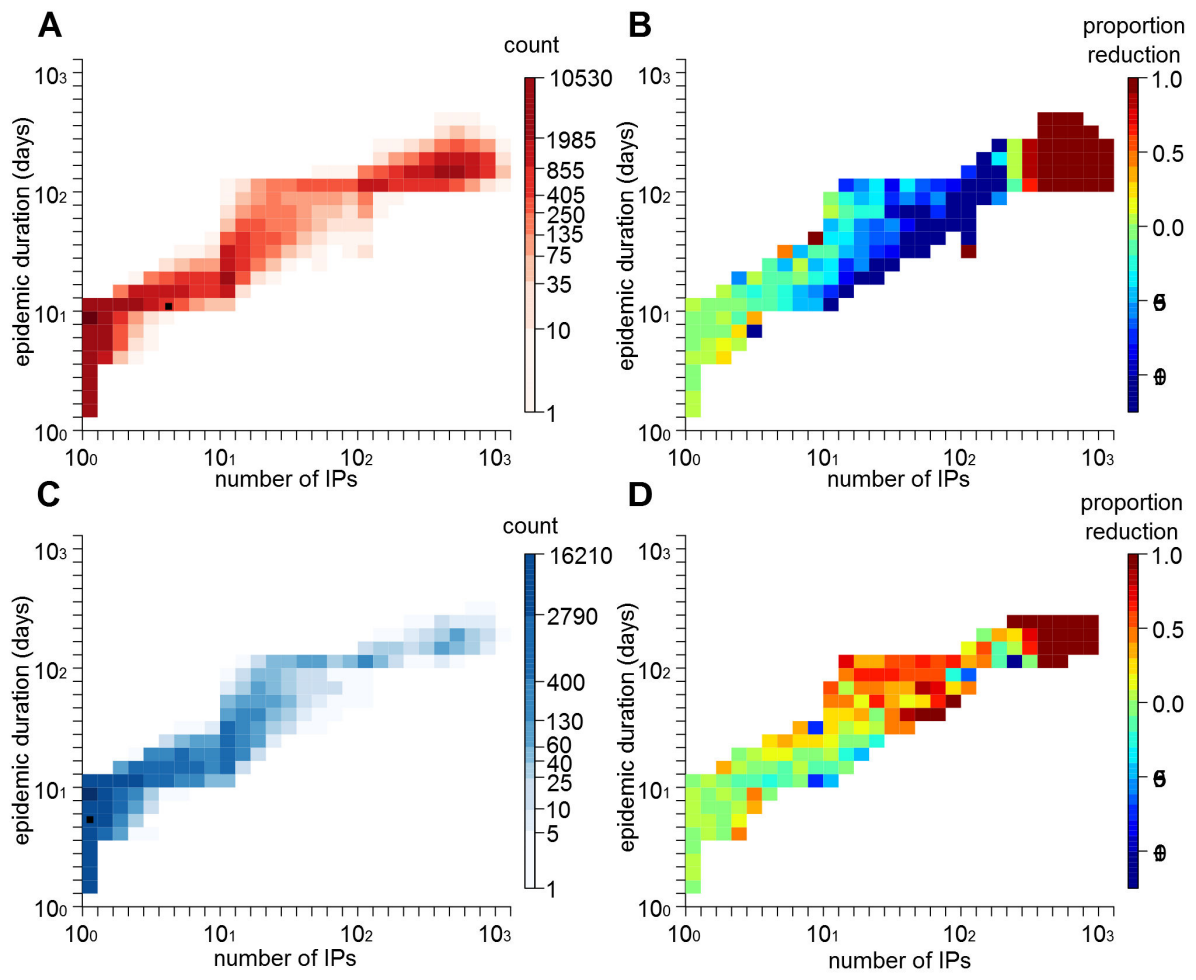
Epidemiologic impacts of a single FMD incursion when routine control measures are implemented alone or together with vaccination. A and C show the number of premises infected (IP) in a given epidemic generated from a single incursion in Southern (A) and Northern (B) counties, versus the number of days that epidemic would last. B and D show the the proportion reduction in the number of epidemics when vaccination is implemented in the field at day 7 and for incursions in Southern (B) and Northern (D) counties. Black rectangle in A and C marks the geometric mean of the number of IPs and epidemic duration for all epidemics generated from incursions in the Southern or Northern counties, respectively. Negative values in B and C indicate an increase in the number of epidemics compared to when no vaccination is implemented, while positive values indicate a decrease in the number of epidemics.

When considering the location of the incursion, Scotland may be divided into two areas that exhibit relatively distinct epidemic patterns (Figure 2): (i) the area comprising the 14 counties in the south of Scotland (now referred to as “Southern counties”); and (ii) the rest of the country (17 counties, now referred to as “Northern counties”). Vaccination was indeed more effective in preventing FMD spread in the situation where incursion occurred in the Southern counties of Scotland than if it occurred elsewhere (Table 1). Vaccination shows a marked impact on the number of animals that are required to be culled to control a FMD epidemic. When incursion occurs in the Southern counties of Scotland, the implementation of a reactive vaccination strategy would, on average, reduce the number of animals culled for disease control purposes by nearly 1600 animals (32%), including 544 cattle (42%). In contrast, little benefit was recorded if incursions occurred in the Northern counties of Scotland, with about 10 animals (3%) saved. Looking at the benefit of vaccination in more detail, vaccination is however of most value in reducing the extent of severe epidemics regardless of where incursions occurred (Table 1, Figure 3).

**Table 1:** Epidemic impact and vaccination benefit for FMD epidemics occurring in Southern or Northern counties of Scotland.

	**Southern counties**			**Northern counties**		
	**No vaccination**	**Vaccination**	**VB^b^**	**No vaccination**	**Vaccination**	**VB^b^**
Cattle density (in heads per km^2^)	48.3	-	-	12.9	-	-
Sheep density (in heads per km^2^)	170.6	-	-	48.0	-	-
Mean^a^ number of IPs (min-Max)	6.08 (1-1109)	4.30 (1-852)	29.4	1.55 (1-1000)	1.51 (1-592)	2.61
Mean^a^ duration (min - Max) (in days)	17.8 (2-630)	14.9 (2-570)	16.0	7.4 (2-549)	7.3 (2-381)	1.99
Mean^a^ number of animals culled (min-Max) (in thousands of heads)	4.8 (<0.01 - 2500)	3.3 (<0.01 - 1598)	32.2	0.36 (<0.01 - 2241)	0.35 (<0.01 - 1223)	3.05
Mean^a^ number of cattle culled (min-Max) (in thousands of heads)	1.3 (<0.01 - 612)	0.8 (<0.01 - 224)	41.4	0.088 (0.01-568)	0.094 (<0.01 - 170)	6.65
Probability^b^ of >100 IPs	19.0	11.3	-	0.5	0.16	-
Probability^b^ of >60 days	23.5	18.3	-	1.5	0.75	-

a mean numbers are geometric mean numbers

b value expressed in percentage.

Epidemic impact was measured over all epidemics generated from a single infected premise (IP) occurring in each areas of Scotland. As a baseline, the culling of IP/DC premises was implemented to control epidemics. When measuring the benefit of vaccination (VB), vaccination was added at day 7 to routine mitigation procedures.

We conducted a sensitivity analysis to investigate how much the spatial pattern of VB would change when more premises were infected prior to the first FMD report. Although increasing the number of IPs at first report drastically increases the impact of generated epidemics (Figure S1), the observed spatial pattern still remains unchanged. Instead values of VB would be accentuated (Figure S2). Overall, when incursions occurred in the Southern counties and detection was late (i.e. when 5 premises are infected prior first detection), vaccinating cattle showed a marked benefit for Scotland, saving on average 54% (n=99,536) of the animals or 68% (*n*=31,302) of the cattle that were culled for disease purposes, compared to the situation where vaccination was not part of the control strategy. In contrast, implementing a vaccination strategy for epidemics that were introduced in the Northern counties would only show a marginal average benefit (<20%), with less than 850 animals and less than 310 cattle saved, even if FMD was detected late.

**Figure 4:**
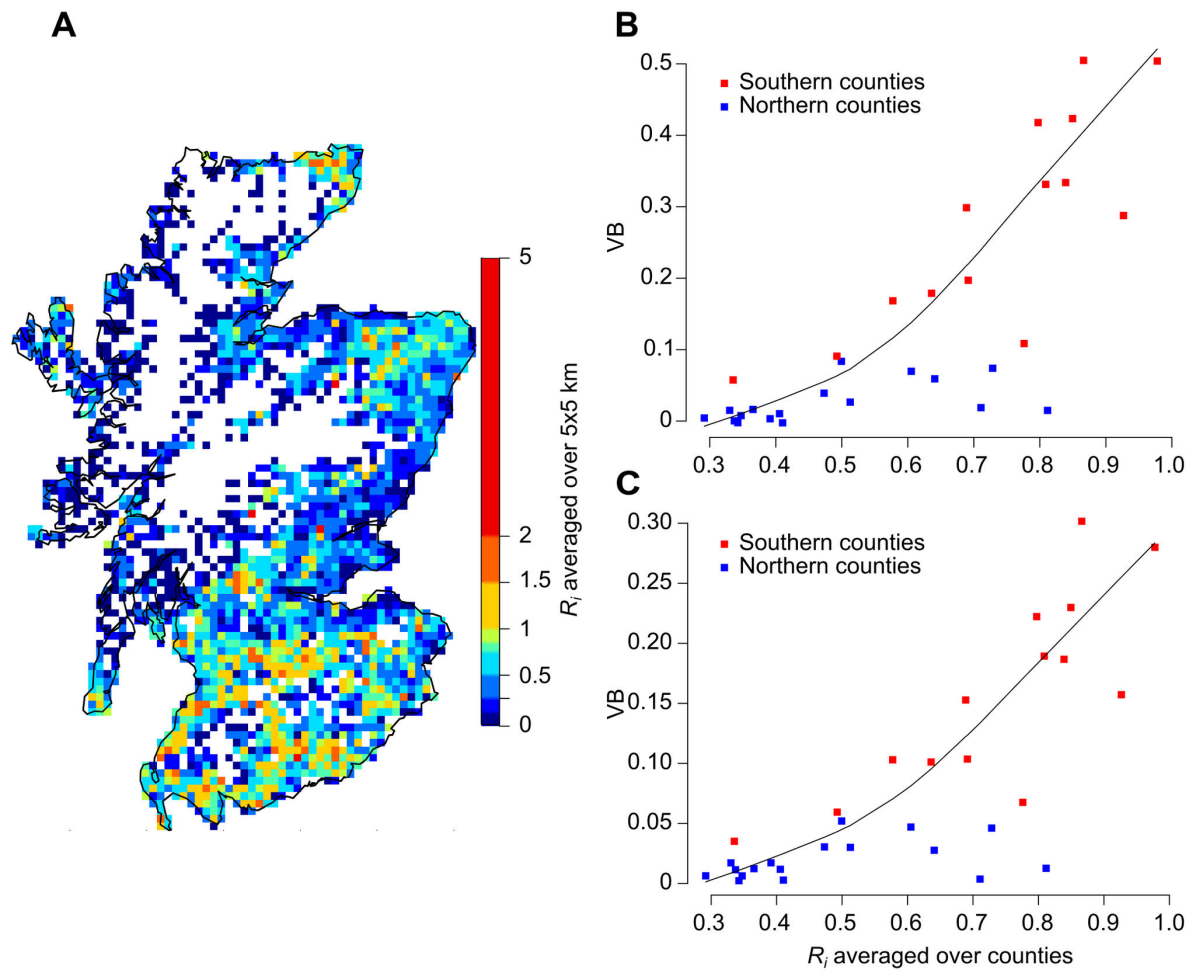
Influence of *R*_*i*_ on the benefit of the vaccination policy. Spatial distribution of the farm-level basic reproductive ratio, *R*_*i*_ (A) and its relation with the FMD vaccination benefit (VB) in Scotland. Vaccination benefit was measured based on the number of infected premises (B) and the epidemic duration (C) in the situation where 1 premise was infected at first detection (early detection). Values of *R*_*i*_ show in B and C are the average across all farms in each county. Note also that county-level estimates in B and C were grouped into the two areas as defined in this study (Figure 2). The colour scale used in the map shows the average value in a 5 × 5 km grid lattice. Estimates of *R*_*i*_ were computed based on the Scottish Agricultural Census June 2011 and using the method described in [15]. Solid curve in B and C represents the smooth fit to better visualize trends in the data set. The fit was generated using the locally weighted scatter plot smoother (LOWESS) method.

Exploring the reasons for such a pattern, we compared the average estimate of VB for epidemics starting in each county with county-level variables informing on the farm industry, namely the cattle and sheep densities (in heads per km^2^) and the number of sheep per head of cattle. We examined also the correlation between VB and the average farm-level basic reproductive ratio *R*_i_ computed as in [15] (Figure 4A). Whilst *R*_*i*_ is correlated with cattle density and sheep density (Spearman rank statistic =0.46 and 0.73), it is also influenced by other factors relating to the underlying herd structure such as production type (i.e. whether herd is cattle only, sheep only, or mixed herd), herd size and density of premises, and to the characteristics of the virus strain (Equations 1-3). Although cattle and sheep densities were significantly correlated with the VB computed based on the number of IPs and duration of epidemics, there was a closer correlation (Spearman rank statistic =0.79 and 0.75), as well as a better fit, between VB and *R*_*i*_ (Figures S3-S4). Figure 4B and 4C show the variation of VB with the number of IPs and the duration of epidemics starting in each county as a function of *R*_*i*_ averaged across all farms present in the county of incursion. Clearly, higher values of *R*_i_ yield higher values of VB.

### Impact of delays in vaccination implementation

**Figure 5:**
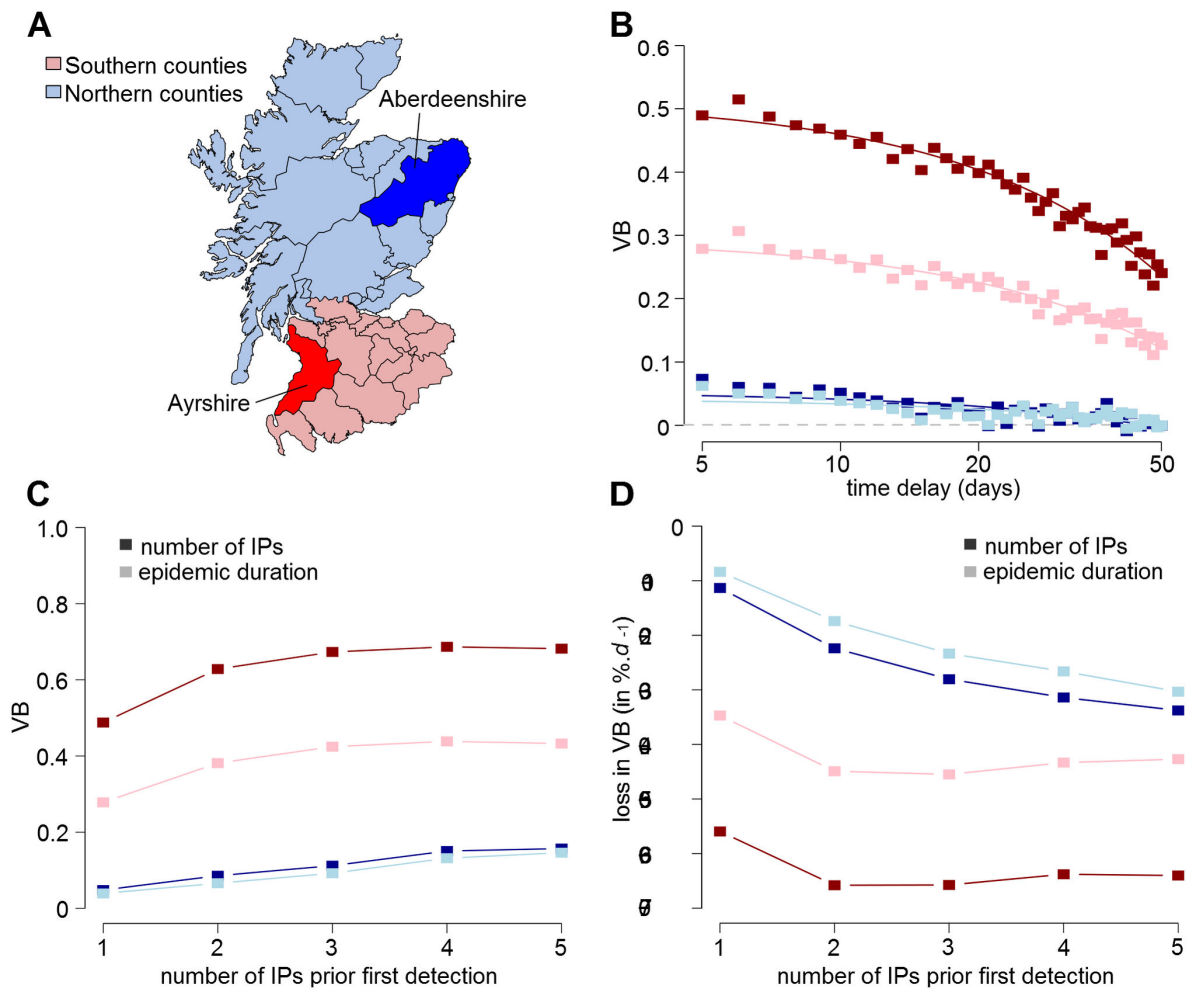
Resilience of vaccination benefit to delays in field implementation. (A) Map showing the location of Aberdeenshire and Ayrshire as examples for the different dynamics identified in Scotland. (B) Effect of delaying the implementation of vaccination in the field on its benefit (VB) for epidemics generated from a single premise in Ayrshire (red) and Aberdeenshire (blue). (C) Risk ratio in VB for each infected premise (IP) increase prior to first detection. (D) Variations in the rate of VB loss for each day increase in delaying its implementation in the field. Values of VB in B, C and D were computed over the number of IP (dark symbols) and the duration (light symbols) of generated epidemics. The probability of severe epidemics was defined as the probability of epidemics either showing >100 IPs (dark symbols) or lasting >60 days (light symbols). Grey dashed line in B indicates the null benefit. Note that x-axis in B are in log-scale.

When investigating the effect of delaying the field implementation of vaccination on its benefit, we restrict our analyses to incursions occurring either in Ayrshire or in Aberdeenshire. These counties were chosen since they are both areas with a high density of premises and animals and represent good examples of the different patterns seen in the southern and northern counties of Scotland (Figure 5A). For both counties, highest VB values were obtained when no delay in implementing the policy occurs (Figure 5B). Obviously this has marked implications when incursions occur in Aberdeenshire, where implementing vaccination shows inherently little benefit. Indeed, waiting 10 days to implement the policy would produce little additional epidemiological benefit for Aberdeenshire incursions, as most epidemics would be finished (Figure 5B, Table 1). Again, vaccination is of most value in reducing the extent of severe epidemics (Figure 3). This means that vaccination is still beneficial for Ayrshire incursions (where the risk of severe epidemics is higher) even if there is a large delay in implementation (Figure 5B).

**Figure 6:**
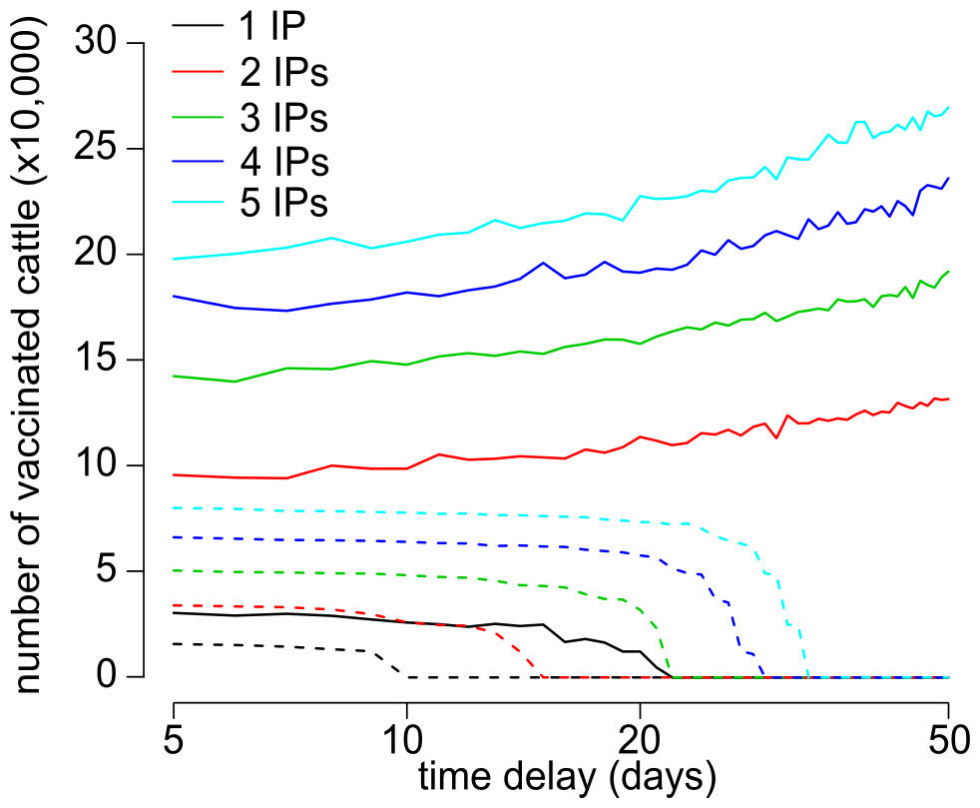
Changes in the number of vaccinated cattle with delays in field implementation. Median number of vaccinated animals (cattle) when controlling a FMD epidemic that were introduced either in Ayrshire (solid line) or Aberdeenshire (dashed line) for increasing time between first detected infected premise (IP) and the field implementation of the vaccination policy and number of infected farms at first detection.

Looking at the effect of late detection, increasing the number of IPs at first report would increase VB (Figure 5C, Figure S5). When five premises are infected at first report, applying vaccination would be about 3.5 and 1.4 times more beneficial than when a single premise is infected in Aberdeenshire and Ayrshire, respectively. However, VB shows a greater sensitivity to delays in the implementation of policy in the field. When the number of IPs prior first to report increases, VB would not only reduce faster for each day increase in delay (Figure 5D) but also would markedly increase the number of cattle which must be vaccinated (Figure 6). Note however that this latter finding is dependent on the location of the incursion. In the situation where FMD is introduced in Aberdeenshire and vaccination is implemented, a stable number of vaccinated animals was generated, on average, that is unaffected by the delay in implementation.

### Sensitivity analysis

**Figure 7:**
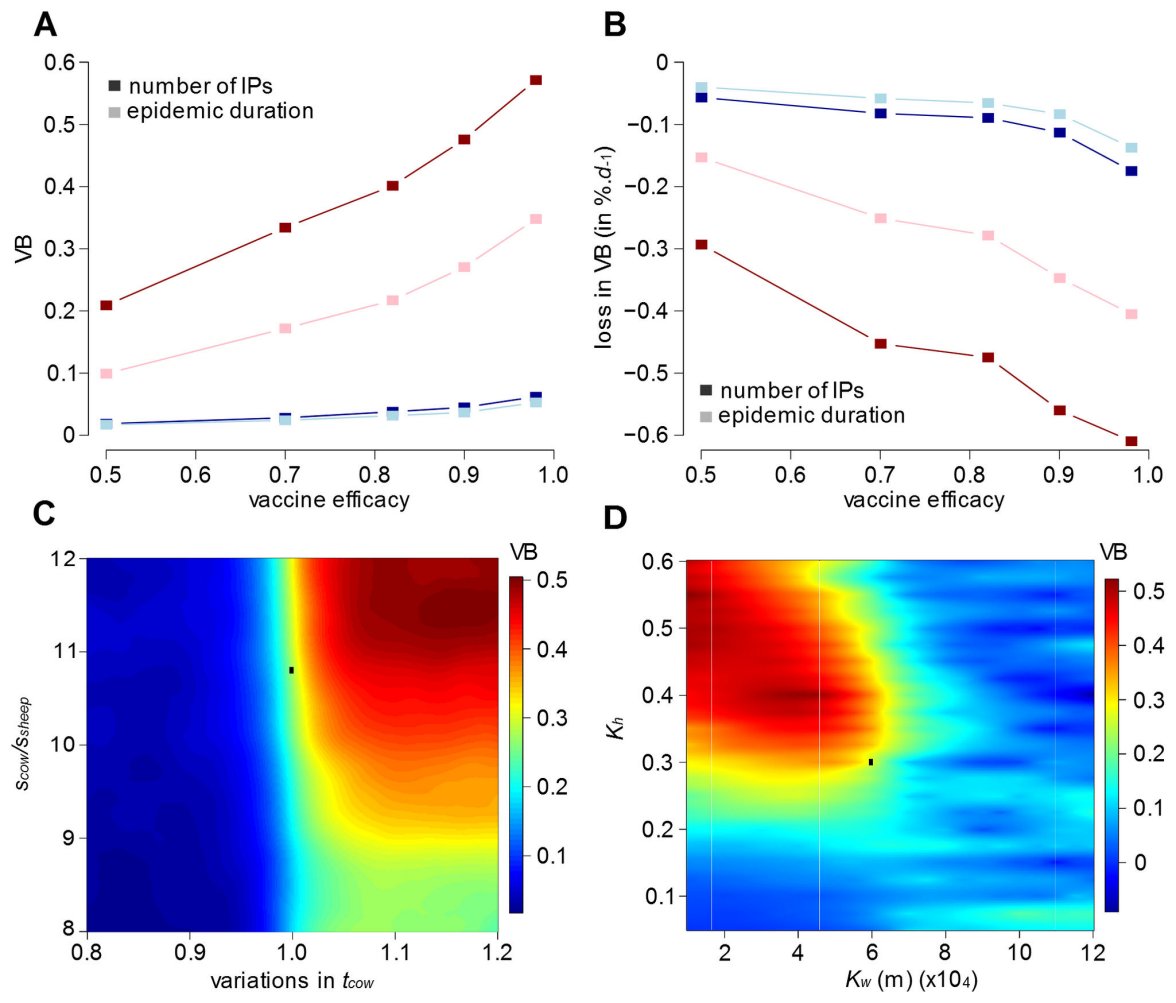
Sensitivity analysis on the benefit of the vaccination policy. Variations in vaccination benefit VB due to changes (A) in vaccine efficacy, (C) in susceptibility (s_cow_) and infectiousness (t_cow_) of cattle, (D) in the width (K_w_) and height (K_h_) of the transmission kernel function. (B) Variations in the loss of VB for each day increases in delaying its implementation in the field. A and B show evolution of the different measures for two counties: Aberdeenshire (red) and Ayrshire (blue) as examples for the different dynamics identified in Scotland (see Figure 5A for details). C and D show evolution of the different measures for Ayrshire as example of the dynamics observed in Scotland. Black rectangle in C and D marks values observed during the 2001 epidemic in Scotland. Simulations in A, C and D were generated considering vaccination policy focused on cattle and implemented at day 7 as a complement to the culling of IP/DC premises.

Figure 7A shows the effect of varying the efficacy of the vaccine between 50% and 98% on VB, based on either the number of infected premises or epidemic duration, when vaccination is implemented at day 7. As previously, the effect of vaccine efficacy on VB is found to be dependent to the location of the initial incursion. In areas such as Ayrshire, the implementation of a vaccination policy shows some benefit in reducing the mean epidemic impact even when the efficacy of the vaccine used is poor. Should an epidemic occur in Aberdeenshire, a vaccination strategy is only beneficial if the vaccine efficacy is high. However, increasing vaccine efficacy makes VB sensitive to delays occurring in the implementation of the policy (Figure S6). Simulations show that loss of VB when an efficacy of 98% is used to control epidemics generated in Ayrshire would be twice as fast as a situation where the vaccine shows 50% efficacy (Figure 7B). As such, even when using a vaccine with high efficacy, to gain maximum benefit any delays in field implementation should be avoided.

In this work, we considered transmission processes similar to what was encountered during the 2001 FMD epidemic in the UK. Departing from this assumption, we tested how VB would change by varying the degree of susceptibility and infectiousness of the target species (here cattle) to FMD virus as well as the shape of the transmission kernel function (in term of width *K*_*w*_ and height *K*_*h*_). Figure 7C and S7 show that when the vaccination target species shows lower susceptibility and infectiousness, a targeted vaccination strategy would quickly lose most of its epidemiologic advantage. In contrast, varying the infectiousness of non-target species (here sheep), up to a situation where they are 30% more infectious than cattle, did not influence our results (Figure S8). Although the response to variations in susceptibility and infectiousness of cattle or sheep was similar between epidemics generated in Aberdeenshire and Ayrshire, the scale of variations were substantially different. Indeed, the range of VB in Aberdeenshire was >5 times smaller than the one estimated in Ayrshire. This indicates that vaccination strategies restricted to incursions in the southern counties of Scotland would not be as advantageous if the circulating strain was either less cattle-specific or more sheep-specific.

Finally, the benefit of vaccinating cattle at day 7 for a wide range of *K*_*h*_ and *K*_*w*_ values is shown in Figure 7D and S9. For low values of *K*_*h*_, little VB was obtained, indicating that mitigation measures restricted to routine procedures would be sufficient to control epidemics. However, when *K*_*h*_ reached a certain critical value, implementing vaccination together with routine mitigation procedures became more beneficial. Definition of this threshold depends on the main aim of the control operations: if the main priority is to limit the number of IPs instead of the duration of the epidemic, vaccination may be implemented at a lower value of *K*_*h*_ if the spatial extent (*K*_*w*_) of the transmission function is large. In all cases however, marked benefit (reduction of >10% in the number of IP and epidemic duration) was found when implementing a vaccination strategy in the situation where the FMD virus strain is characterised by a high local spread (*K*_*h*_ ≥0.3) and limited spatial extent (*K*_*w*_ ≤70km). It should be noted that we restricted vaccination to premises located within a 10km radius around IPs, as regulated in Scotland. Varying the size of the ring would most likely change the spatial extent threshold at which vaccination is beneficial. Therefore, such a threshold should be considered in general terms rather than as absolute values.

## Discussion

The use of vaccination in FMD control is a potentially valuable tool but its implementation remains a contentious issue. Despite the relatively large scientific literature on the potential benefits of vaccination, little consensus exists on when vaccination is most beneficial, and hence what factors might trigger its implementation as part of a FMD control strategy [9]. This lack of consensus and clarity means control managers remain unclear on when vaccination could be used. This may cause delays in decision making, which could, in itself, potentially affect the efficiency of the strategy. Starting from this observation, we explored the influence of different putative drivers on the effectiveness of a vaccination-to-live strategy for FMD. In particular, we tested whether changes in the delay of implementation and the location of the incursion would affect the epidemiological benefit of such a strategy.

In this paper, we found that the celerity at which vaccination is implemented in the field is critical for the effectiveness of the policy. For instance, every day increase in delaying its implementation would linearly reduce its effectiveness, not only increasing the number of IPs, the duration of epidemics and the number of animals culled for disease control purposes, but also the number of vaccinated animals. The likelihood of severe epidemics (here, defined as epidemics with >100 IPs and lasting for >60 days) would also be reduced if the policy is implemented rapidly. While these findings are consistent with other simulation outputs carried out in a German context [8], we further show that the rate at which benefit is lost strongly depends on the location of the incursion as well as the efficacy of the vaccination available and extent of the silent spread (in terms of number of premises infected prior to first detection). The reasons for such effects are unclear but it is most likely related to the initial benefit in implementing vaccination. In other words, greater the benefit when vaccinating rapidly, faster is the loss when delays occur. However, we also showed that waiting 10 days to implement the policy would produce little additional epidemiological difference for Aberdeenshire incursions, as most epidemics would be finished. This result indicates that, in areas where vaccination is most often of little benefit (such as in Aberdeenshire), delaying the implementation of vaccination in the field may be of limited epidemiological cost as long as the epidemic does not spread to areas where the risk of severe epidemics is higher (such as in Southern counties of Scotland).

Whilst we considered the impact of vaccination in terms of the size and duration of FMD epidemics, another potential effect of vaccination is the possible development of carrier animals that remain persistently infected and a potential source of infection for further outbreaks. The risk of vaccinated farms having persistent carrier animals is related to the number of infected animals that already exist before the total immunity of the herd is reached [24,25]. Allowing FMD to spread prior to implementing vaccination is likely to increase the probability that vaccinated premises would be exposed to infection. This means that delaying the implementation of a vaccination programme would not only reduce its epidemiological benefit but also pose a significant risk on rapidly regaining FMD-free status. This stresses further the importance of implementing vaccination in the field rapidly.

Beyond the effect of delays in its implementation, the epidemiological advantage of a reactive vaccination strategy is also sensitive to the conditions of the incursion at hand. Although we tested numerous scenarios, two main issues influence the epidemiological benefit of deploying vaccination in the field: the location of the incursion, and the virus characteristics of the circulating strain.

For Scotland, deploying a reactive vaccination strategy at the end of the first week would save the slaughter of nearly a third of the animals if incursion occurs in the southern counties, when comparing with a no vaccination strategy. In contrast, not much difference was observed if incursion occurs in the rest of the country. However, the model did not consider cross border spread of FMD, since only farms present within the boundaries of Scotland were considered. Ignoring the epidemiological impact of incursion events located near the border with England would both limit the number of premises susceptible to be infected and not account for re-incursion events from England. These would most likely underestimate the benefit of vaccination in areas near the border and make our results conservative. Such a result is however not irrelevant as it represents a basis to better stratify the implementation of vaccination in Scotland. Applying vaccination when incursion events occur strictly in these high-risk (Southern) counties would, indeed, avoid unnecessary cost. Livestock density is widely believed to be the main driver for such spatial pattern [7–9,26–28] and, indeed, the density of susceptible animals happens to markedly differ between these high-risk counties and the rest of the country. In particular, there are nearly 4 times more cattle and sheep in the Southern counties than for the rest of the country (Table 1). However, livestock density alone does not predict the susceptibility of premises to be infected nor their infectiousness [27,28], and thereby transmission. Our results were consistent with this, and suggest that the spatial variation in VB is inherently related to the spatial variation in the farm-level reproduction value, *R*_*i*_ (Figure 4, S3 and S4), rather than simply being due to livestock density. Indeed, *R*_*i*_ is not only influenced by the underlying local population density and structure (such as production type, herd size and density of premises), but also the characteristics of the virus strain (Equations 1-3). Given that *R*_*i*_ appears to be the main driver of vaccination benefit, simply identifying areas of highest population density may not capture all complexities that influence the spread of disease as well as the benefit of implementing vaccination, so efforts should be carried out to estimate *R*_*i*_ within a time-frame allowing the stratification of control activities, should FMD be introduced.

An important problem when estimating *R*_*i*_ is that it depends on the intrinsic characteristics of the circulating virus; notably because of its influence on the susceptibility and the transmissibility of each premise to infection, and on the transmission kernel function. Following the assumption that the epidemiological advantage of mitigation measures is more likely to depend on the shape of the transmission kernel function than on other virus characteristics [29], we first explored the effect of the intrinsic characteristics of the virus on the benefit of implementing a vaccination policy by varying the transmission kernel function. It was apparent that the kernel shape used as baseline (i.e. the transmission kernel function defined during the 2001 UK FMD epidemic) was not optimal for the implementation of vaccination. Instead, reactive vaccination would have been increasingly more advantageous if the circulating FMD virus strain shows a greater propensity to spread locally.

In the event of a real incursion, the serotype and strain of the FMD virus responsible of the incursion would be known fairly quickly [30]. However, the characteristics of this strain and hence the definition of the kernel shape remain dependent on the amount of information field veterinarians may collect, or the degree of prior knowledge available for a particular strain type. Although recent studies have described methods to estimate the shape of the transmission kernel function using real-time outbreak data [31,32], it is still likely to take some time before parameters can be estimated accurately. As seen above, delaying the implementation of vaccination reduces the benefit of a reactive vaccination strategy. As argued elsewhere [33], it is therefore important that qualitative rules should be generated to inform policy within the early stages of an epidemic until sufficient data are collected to enable full quantitative assessment of the transmission kernel function.

Another factor that needs to be considered when deciding if vaccination is worth implementing is the importance of restricting vaccination to target species. During the 2001 FMD epidemic in the UK, large cattle herds were critical for the spread of FMD due to their greater susceptibility to infection and infectiousness [27,28]. Consequently, vaccinating only premises with large cattle herds increased the effectiveness of reactive ring vaccination [11,24,34]. Such a strategy is now currently part of the contingency plan [23] and was therefore our baseline in this study. We showed that when the vaccination target species show less susceptibility and infectiousness than the baseline, a targeted vaccination strategy would quickly lose most of its epidemiologic advantage. It is however important to note that we considered that FMD would only circulate within the cattle and sheep industry. Should a greater involvement of pigs and/or substantial airborne spread occur, a different epidemiological picture would emerge and targeting species for vaccination would need to be reconsidered.

In this paper, we defined the benefit of a vaccination strategy based on the number of animals, premises, and days saved by the implementation of vaccination. However, the benefit associated with vaccination in term of animals saved needs to be counterbalanced against the extra costs related to its implementation in the field. Notably, costs directly related to the control activities should be balanced with those associated with export restrictions, variations in market prices and diminished future production [5,35]. Given the temporal variations in the effect(s) of these latter factors, vaccination would not always be economically beneficial, even when it appears to be epidemiologically beneficial. As such, considering the economic impact of epidemics, along with its epidemiology, may affect the time-window within which the policy is cost-efficient. In particular, short and small epidemics may be most penalised economically from the additional trade restrictions induced by vaccination. Consequently, if a decision to vaccinate is made, vaccination must be implemented quickly to optimise its overall benefit.

In conclusion, we explored the effect of several factors that influence the benefits of implementing a reactive vaccination-to-live policy when facing epidemics of infectious disease such as FMD in Scotland. We have shown that the decision to vaccinate, or not, is not straightforward and strongly depends on the spatial variation in the farm-level basic reproductive ratio values *R*_*i*_, illustrated here by the differences between the southern and northern counties of Scotland. However, if a decision to vaccinate is made, we have shown that delaying its implementation in the field may markedly reduce its benefit.

## Supporting Information

Figure S1 **Epidemic impact of a FMD incursion in Scotland.** Violin plots showing the distribution of (A) the number of infected premises, (B) epidemic duration, (C) the number of animals culled and (D) the number of cattle culled for increasing number of infected premises at first detection and for all epidemics initiated in either Southern or Northern counties. White circles and squares represent the geometric mean of each distribution for Southern or Northern counties, respectively.
(TIF)

Figure S2 **Benefit of vaccinating cattle.** Changes in the benefit of vaccination (VB) in term of number of animal culled for disease control purposes (i.e. proportion of animals saved) when varying the number of infected farms at time of detection: 1 IP (top left), 2 IPs (top right), 3IPs (bottom left) and 5 IPs (bottom right). Simulations were generated considering that a vaccination policy would be implemented in the field at day 7 as a complement to the culling of IP/DC premises.
(TIF)

Figure S3 **Influence of livestock densities and *R*_*i*_ on the benefit of the vaccination policy on reducing the number of infected premises.** Relationships (lower panel) and correlation (upper panel) between the values of the vaccination benefit (VB), the farm-level basic reproduction number *R*_*i*_, cattle and sheep animal densities and the cattle/sheep ratio computed for each county. Correlations between variables were estimated using the Spearman rank statistics, and those with a correlation coefficient above the absolute value of 0.7 were considered as correlated. Numbers and stars in the upper panel indicates the Spearman rank statistics and the associated significant level such as (*) P<0.05, (**) P<0.01 and (***) P<0.001. Dots in the lower panel are county-level estimates, grouped into the two areas as defined in this study (Figure 2). Values of VB were computed upon the mean number of infected premises (IPs) and considered that a single premise is infected at first detection (early detection). Values of *R*_*i*_ were computed by averaging the farm-level *R*_*i*_ across all farms in each counties. Estimates of *R*_*i*_ were computed based on the Scottish Agricultural Census June 2011 and using the method described in [15].
(TIF)

Figure S4 **Influence of livestock densities and *R*_*i*_ on the benefit of the vaccination policy on reducing the epidemic duration.** Relationships (lower panel) and correlation (upper panel) between the values of the vaccination benefit (VB), the farm-level basic reproduction number *R*_*i*_, cattle and sheep animal densities and the cattle/sheep ratio computed for each counties. Correlations between variables were estimated using the Spearman rank statistics, and those with a correlation coefficient above the absolute value of 0.7 were considered as correlated. Numbers and stars in the upper panel indicates the Spearman rank statistics and the associated significant level such as (*) P<0.05, (**) P<0.01 and (***) P<0.001. Dots in the lower panel are county-level estimates, grouped into the two areas as defined in this study (Figure 2). Values of VB were computed upon the mean epidemic duration and considered that a single premise is infected at first detection (early detection). Values of *R*_*i*_ were computed by averaging the farm-level *R*_*i*_ across all farms in each counties. Estimates of *R*_*i*_ were computed based on the Scottish Agricultural Census June 2011 and using the method described in [15].
(TIF)

Figure S5 **Changes in vaccination benefit for an increasing implementation delay and number of infected premises prior detection.** Vaccination benefit (VB) was measured based on the geometric mean of (A) infected premises (IPs), (B) epidemic duration, (C) number of animals culled and (D) number of cattle culled during control operations. Figures show evolution of the different measures for two counties: Ayrshire (solid line) and Aberdeenshire (dashed line) as examples for the different dynamics identified in Scotland. Grey dashed line indicates the null benefit.
(TIF)

Figure S6 **Changes in vaccination benefit for an increasing implementation delay and vaccine efficacy.** Vaccination benefit (VB) was measured based on the geometric mean of (A) infected premises, (B) epidemic duration, (C) number of animals culled and (D) number of cattle culled during control operations. Figures show evolution of the different measures for two counties: Ayrshire (solid line) and Aberdeenshire (dashed line) as examples for the different dynamics identified in Scotland. Grey dashed line indicates the null benefit.
(TIF)

Figure S7 **Changes in vaccination benefit for an increasing cattle-specific susceptibility and transmissibility.** Smoothed image plot showing the changes in vaccination benefit (VB) in term of (A, C) infected premises and (B, D) epidemic duration when varying the cattle-specific susceptibility *s*_*cow*_ and transmissibility *t*_*cow*_. Simulations were generated considering a single infected premise at first detection and vaccinating cattle at day 7 as a complement to the culling of IP/DC premises. Figures show the evolution of the different measures for two counties: (A-B) Aberdeenshire and (C-D) Ayrshire as examples for the different dynamics identified in Scotland. The black circle shows the point at which *scow*/*s*_*sheep*_ and *t*_*cow*_ take the Scotland-specific values fitted from the 2001 epidemic.
(TIF)

Figure S8 **Changes in vaccination benefit for an increasing cattle-specific susceptibility and sheep-specific transmissibility.** Smoothed image plot showing the changes in vaccination benefit (VB) in term of (A, C) infected premises and (B, D) epidemic duration when varying the cattle-specific susceptibility *s*_*cow*_ and sheep-specific transmissibility *t*_*sheep*_. Simulations were generated considering a single infected premise at first detection and vaccinating cattle at day 7 as a complement to the culling of IP/DC premises. Figures show the evolution of the different measures for two counties: (A-B) Aberdeenshire and (C-D) Ayrshire as examples for the different dynamics identified in Scotland. The black circle shows the point at which *scow*/*s*_*sheep*_ and *tsheep*/*t*_*cow*_ take the Scotland-specific values fitted from the 2001 epidemic.
(TIF)

Figure S9 **Changes in vaccination benefit (VB) when varying the transmission kernel shape.** Smoothed image plot showing the changes in VB in term of infected premises (A, C) and epidemic duration (B, D) when varying the width (*K_w_*) and height (*K_h_*) of the transmission kernel function. Simulations were generated considering a single infected premise at first detection and vaccinating cattle at day 7 as a complement to the culling of IP/DC premises. Figures show evolution of the different measures for two counties: (A, B) Aberdeenshire and (C, D) Ayrshire as examples for the different dynamics identified in Scotland. The black circle shows the point at which *K*_*w*_ and *K*_*h*_ take the values observed during the 2001 epidemic.
(TIF)

Table S1 **Farm data for 2011 in Scotland and used in the model.**
(DOC)
