# Supplementary figures and images for: Vaccination against Foot-And-Mouth Disease: Do Initial Conditions Affect Its Benefit?
Source: PLoS One. 2013 Oct 4;8(10):e77616. doi: 10.1371/journal.pone.0077616 (PMC3815046; doi:10.1371/journal.pone.0077616)

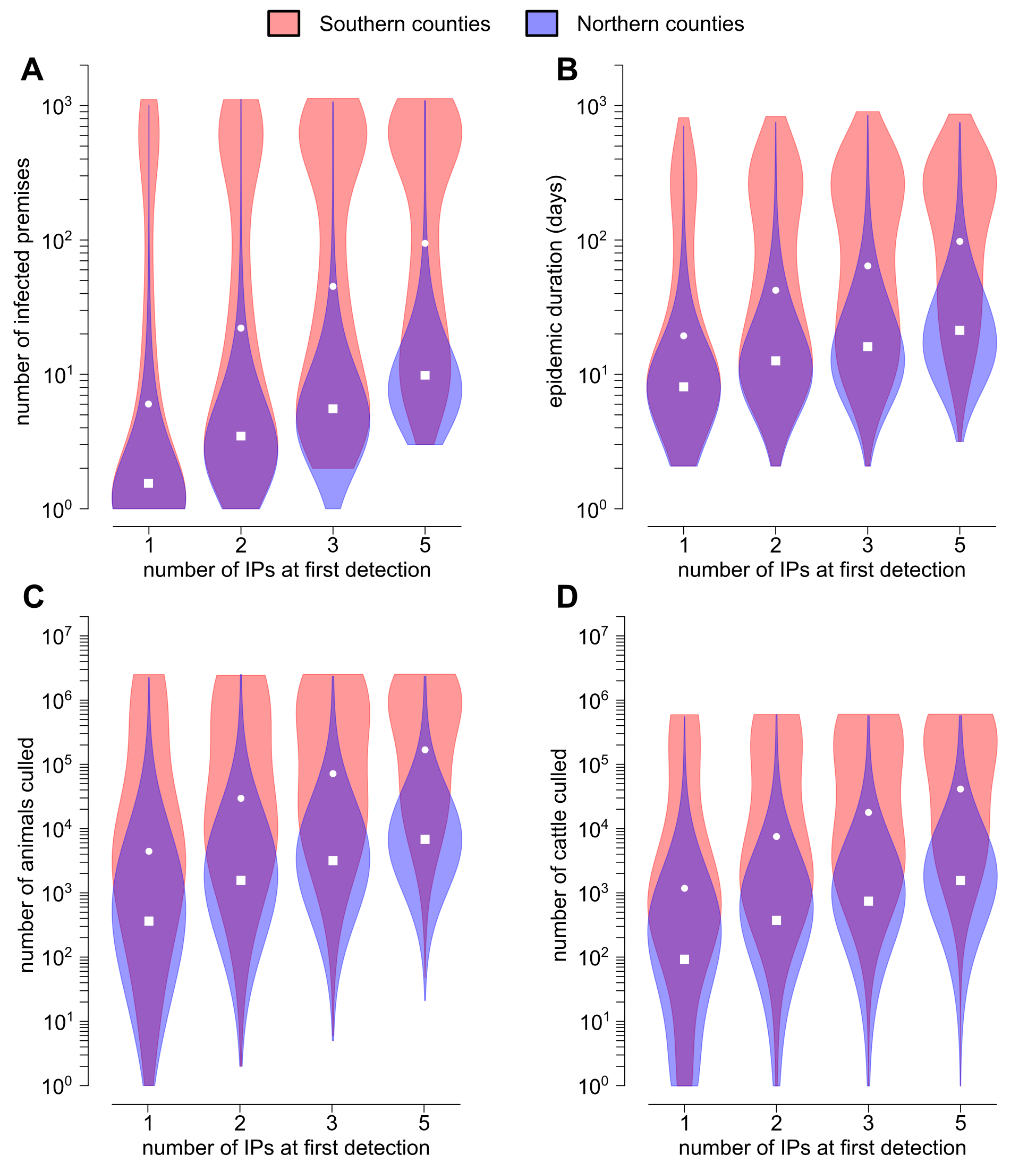

Supplement: Figure S1 — Epidemic impact of a FMD incursion in Scotland. Violin plots showing the distribution of (A) the number of infected premises, (B) epidemic duration, (C) the number of animals culled and (D) the number of cattle culled for increasing number of infected premises at first detection and for all epidemics initiated in either Southern or Northern counties. White circles and squares represent the geometric mean of each distribution for Southern or Northern counties, respectively. (TIF) [file pone.0077616.s001.tif]

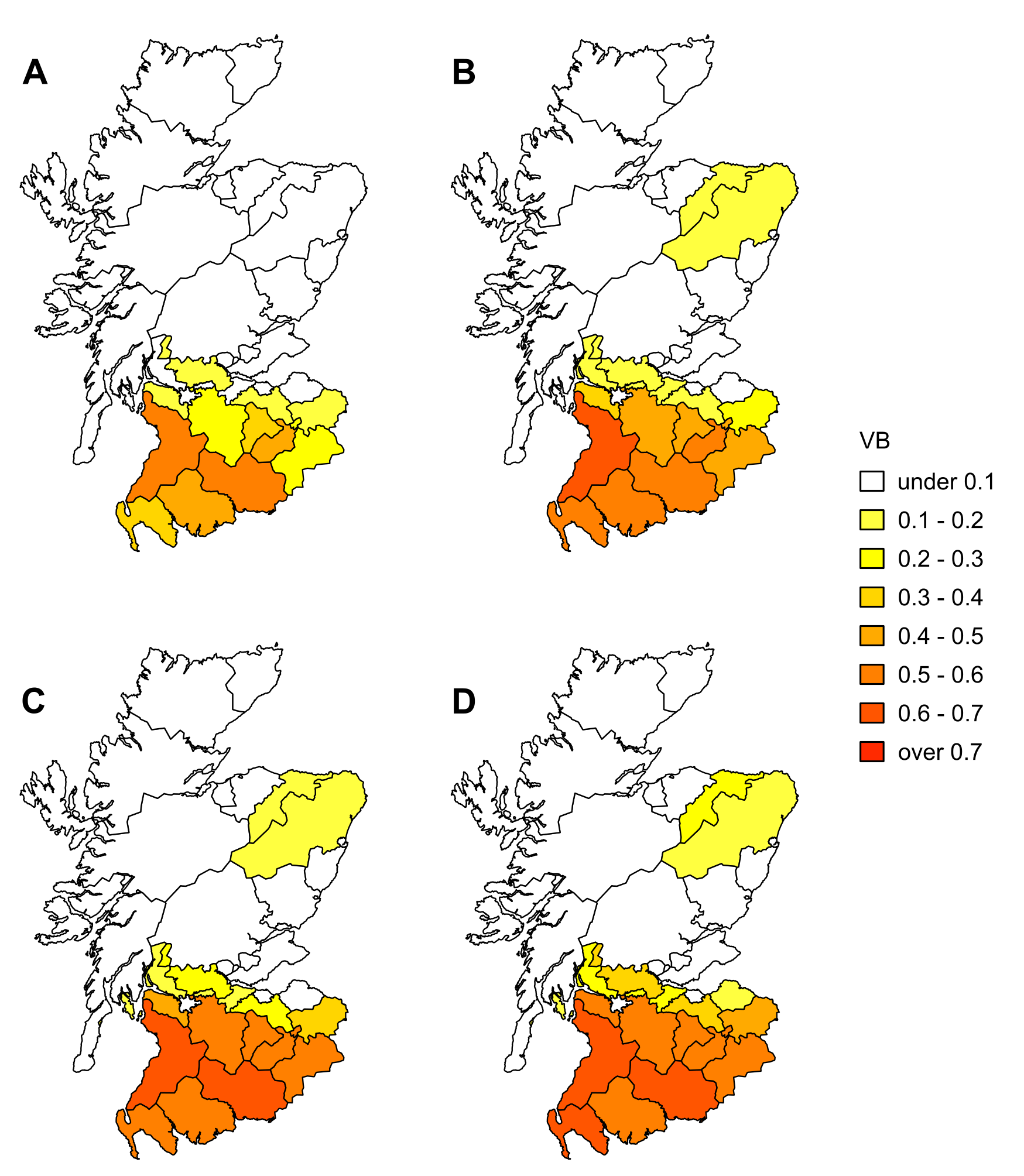

Supplement: Figure S2 — Benefit of vaccinating cattle. Changes in the benefit of vaccination (VB) in term of number of animal culled for disease control purposes (i.e. proportion of animals saved) when varying the number of infected farms at time of detection: 1 IP (top left), 2 IPs (top right), 3IPs (bottom left) and 5 IPs (bottom right). Simulations were generated considering that a vaccination policy would be implemented in the field at day 7 as a complement to the culling of IP/DC premises. (TIF) [file pone.0077616.s002.tif]

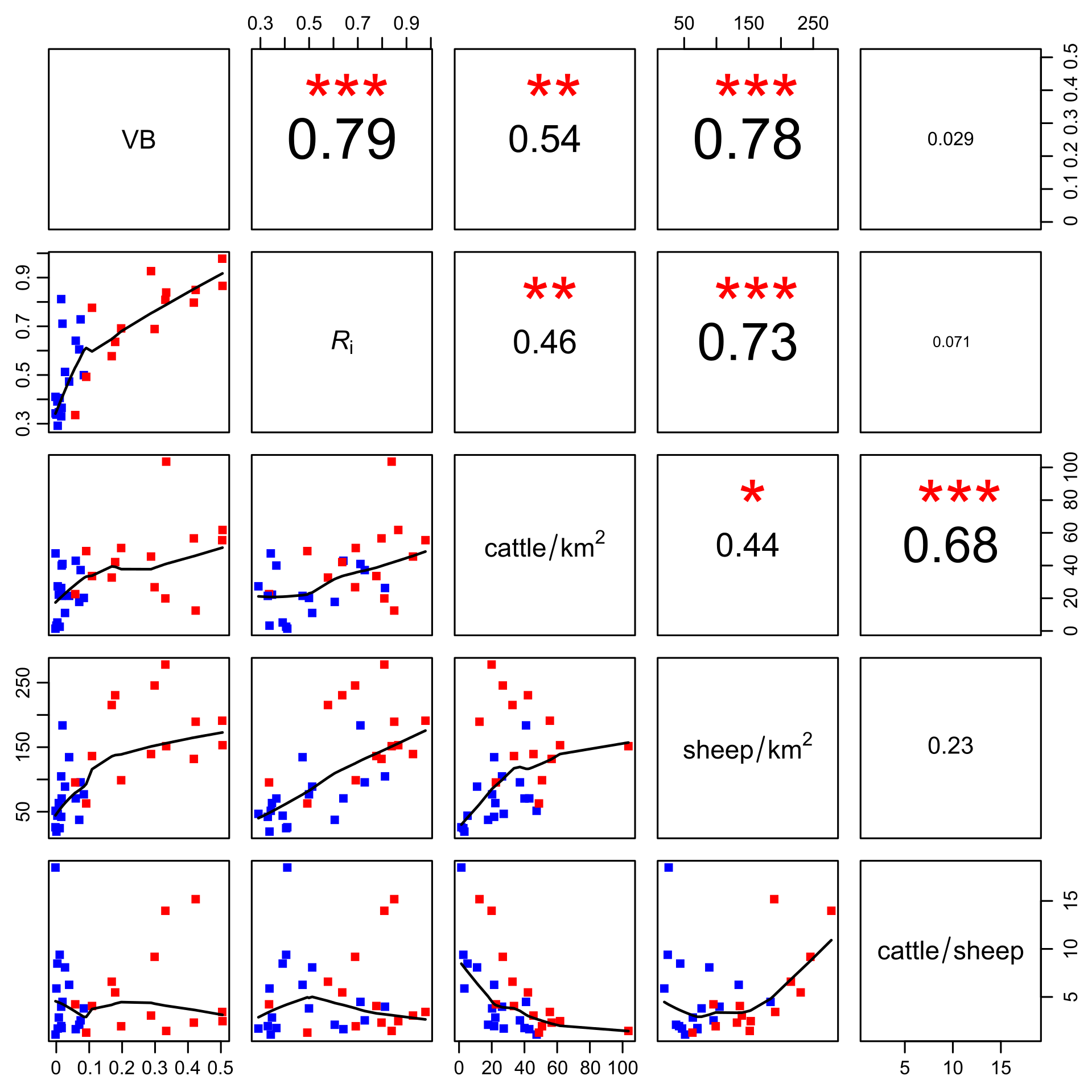

Supplement: Figure S3 — Influence of livestock densities and Ri on the benefit of the vaccination policy on reducing the number of infected premises. Relationships (lower panel) and correlation (upper panel) between the values of the vaccination benefit (VB), the farm-level basic reproduction number R i, cattle and sheep animal densities and the cattle/sheep ratio computed for each county. Correlations between variables were estimated using the Spearman rank statistics, and those with a correlation coefficient above the absolute value of 0.7 were considered as correlated. Numbers and stars in the upper panel indicates the Spearman rank statistics and the associated significant level such as (*) P<0.05, (**) P<0.01 and (***) P<0.001. Dots in the lower panel are county-level estimates, grouped into the two areas as defined in this study (Figure 2). Values of VB were computed upon the mean number of infected premises (IPs) and considered that a single premise is infected at first detection (early detection). Values of R i were computed by averaging the farm-level R i across all farms in each counties. Estimates of R i were computed based on the Scottish Agricultural Census June 2011 and using the method described in [15]. (TIF) [file pone.0077616.s003.tif]

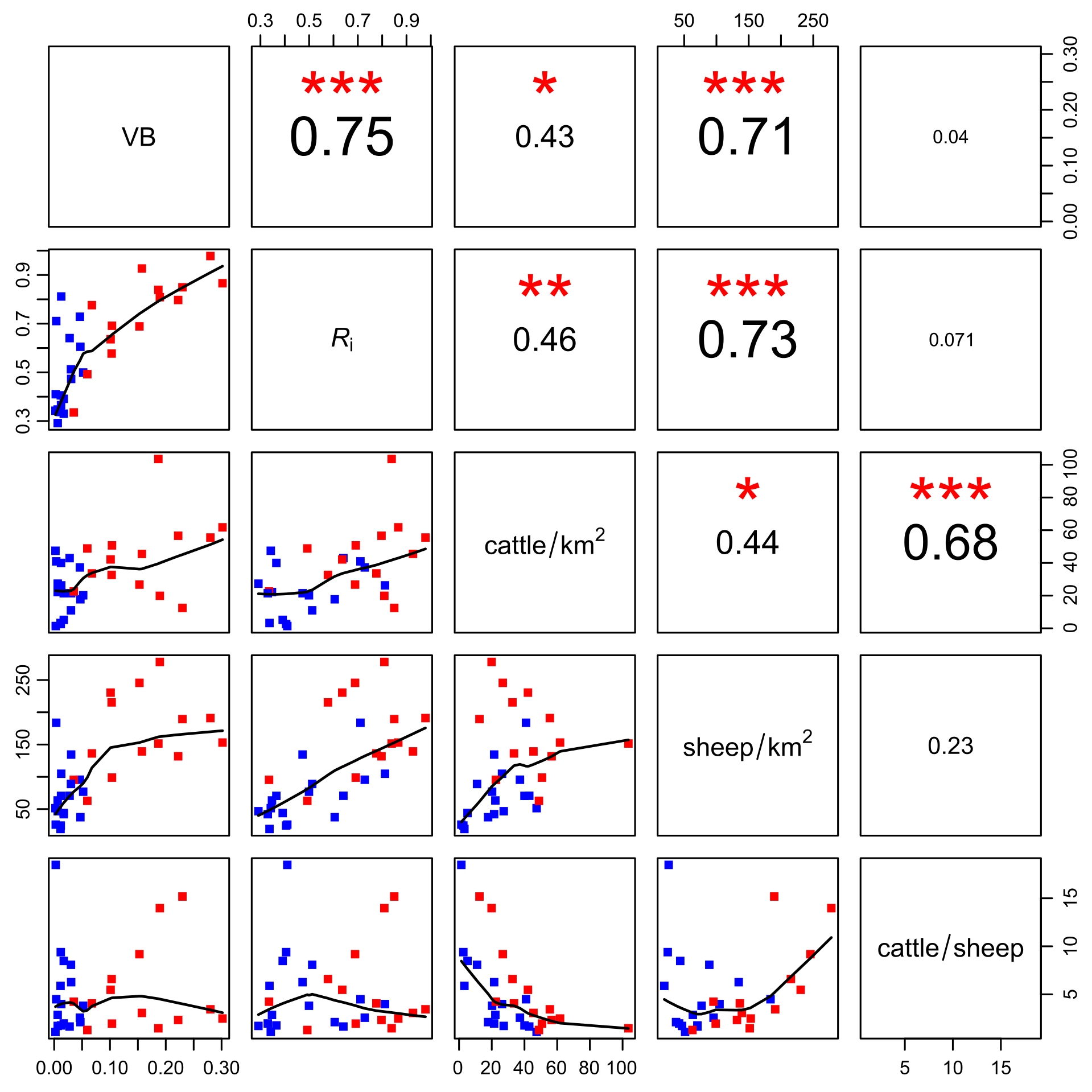

Supplement: Figure S4 — Influence of livestock densities and Ri on the benefit of the vaccination policy on reducing the epidemic duration. Relationships (lower panel) and correlation (upper panel) between the values of the vaccination benefit (VB), the farm-level basic reproduction number R i, cattle and sheep animal densities and the cattle/sheep ratio computed for each counties. Correlations between variables were estimated using the Spearman rank statistics, and those with a correlation coefficient above the absolute value of 0.7 were considered as correlated. Numbers and stars in the upper panel indicates the Spearman rank statistics and the associated significant level such as (*) P<0.05, (**) P<0.01 and (***) P<0.001. Dots in the lower panel are county-level estimates, grouped into the two areas as defined in this study (Figure 2). Values of VB were computed upon the mean epidemic duration and considered that a single premise is infected at first detection (early detection). Values of R i were computed by averaging the farm-level R i across all farms in each counties. Estimates of R i were computed based on the Scottish Agricultural Census June 2011 and using the method described in [15]. (TIF) [file pone.0077616.s004.tif]

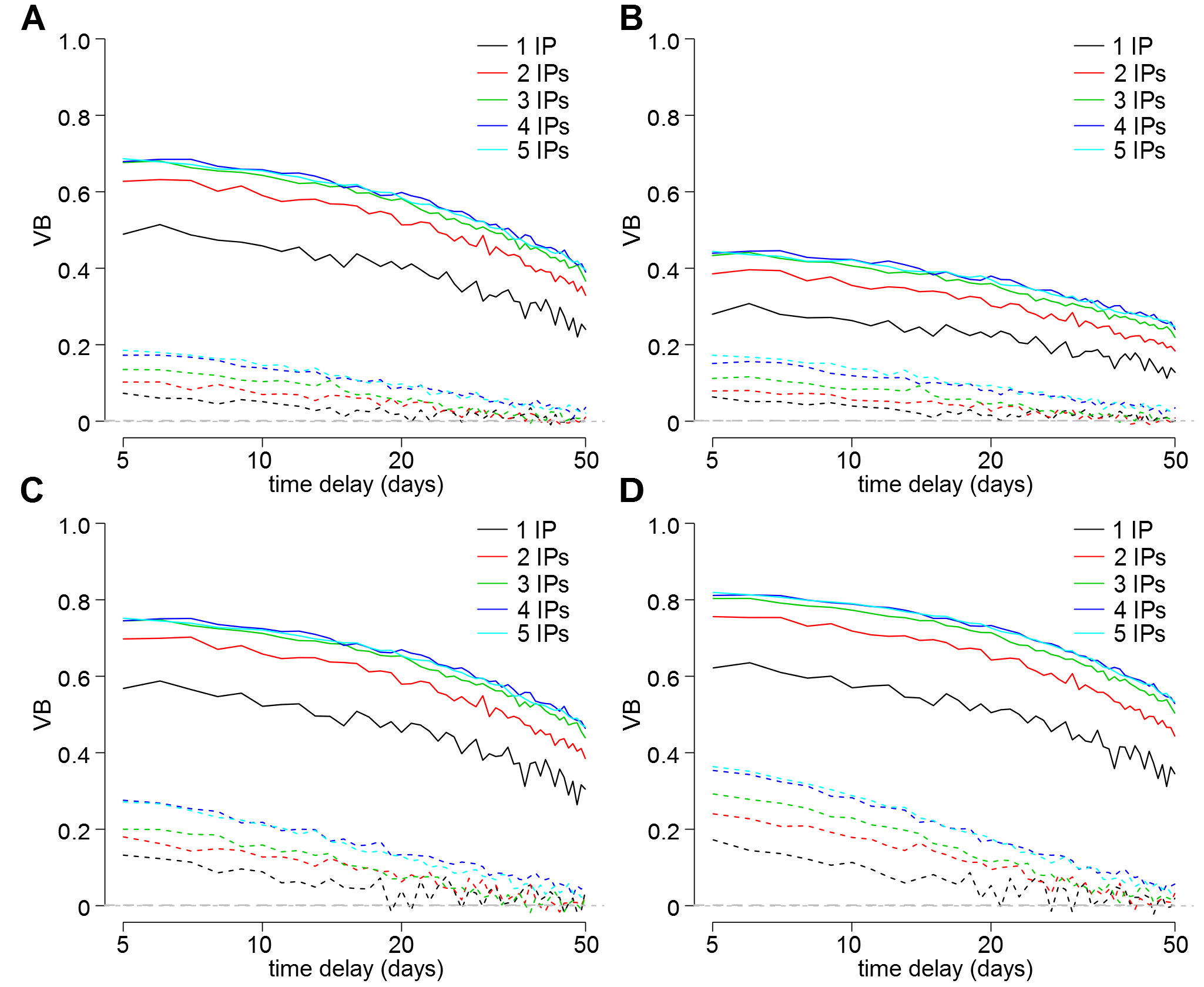

Supplement: Figure S5 — Changes in vaccination benefit for an increasing implementation delay and number of infected premises prior detection. Vaccination benefit (VB) was measured based on the geometric mean of (A) infected premises (IPs), (B) epidemic duration, (C) number of animals culled and (D) number of cattle culled during control operations. Figures show evolution of the different measures for two counties: Ayrshire (solid line) and Aberdeenshire (dashed line) as examples for the different dynamics identified in Scotland. Grey dashed line indicates the null benefit. (TIF) [file pone.0077616.s005.tif]

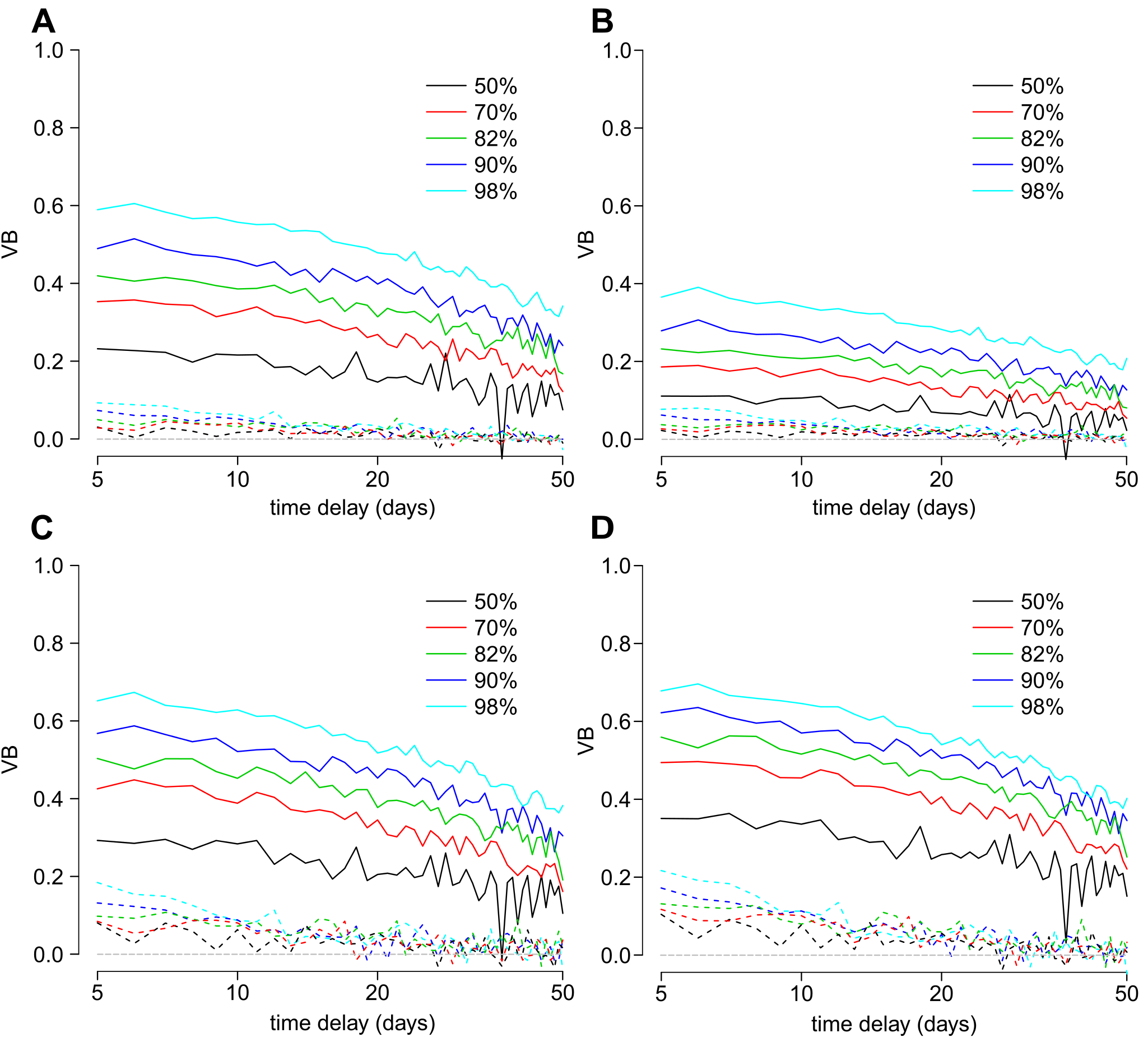

Supplement: Figure S6 — Changes in vaccination benefit for an increasing implementation delay and vaccine efficacy. Vaccination benefit (VB) was measured based on the geometric mean of (A) infected premises, (B) epidemic duration, (C) number of animals culled and (D) number of cattle culled during control operations. Figures show evolution of the different measures for two counties: Ayrshire (solid line) and Aberdeenshire (dashed line) as examples for the different dynamics identified in Scotland. Grey dashed line indicates the null benefit. (TIF) [file pone.0077616.s006.tif]

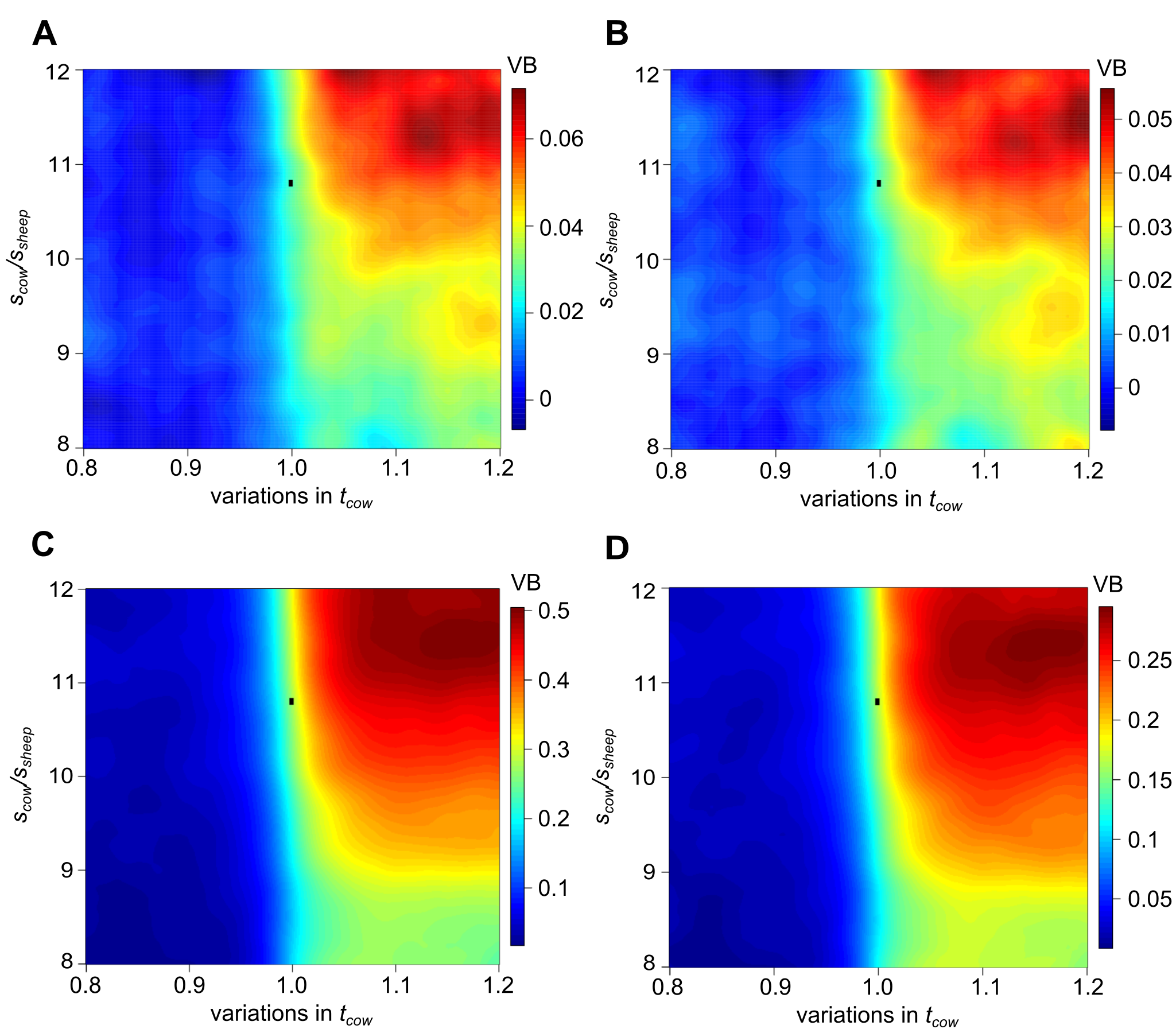

Supplement: Figure S7 — Changes in vaccination benefit for an increasing cattle-specific susceptibility and transmissibility. Smoothed image plot showing the changes in vaccination benefit (VB) in term of (A, C) infected premises and (B, D) epidemic duration when varying the cattle-specific susceptibility s cow and transmissibility t cow. Simulations were generated considering a single infected premise at first detection and vaccinating cattle at day 7 as a complement to the culling of IP/DC premises. Figures show the evolution of the different measures for two counties: (A-B) Aberdeenshire and (C-D) Ayrshire as examples for the different dynamics identified in Scotland. The black circle shows the point at which scow/s sheep and t cow take the Scotland-specific values fitted from the 2001 epidemic. (TIF) [file pone.0077616.s007.tif]

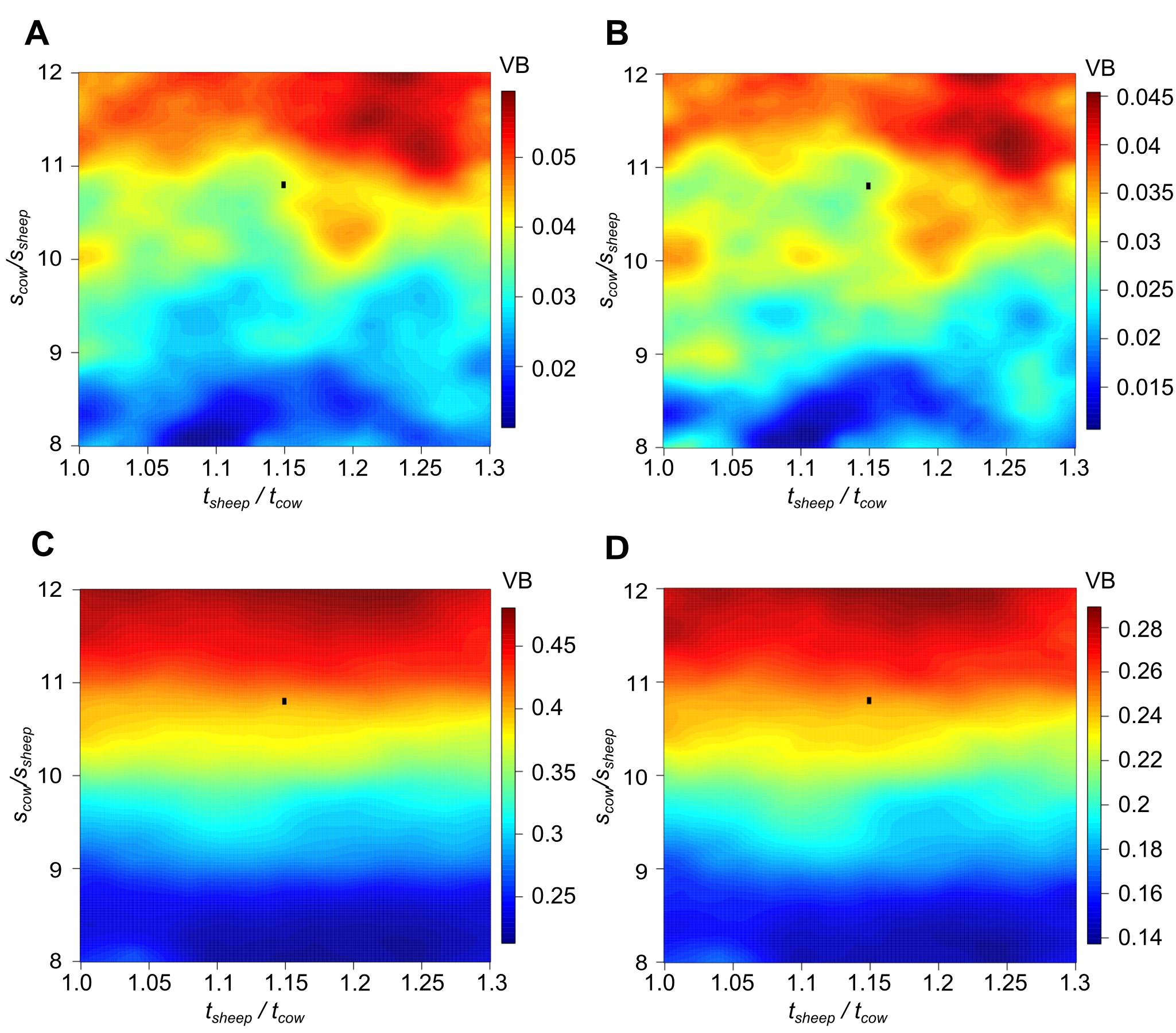

Supplement: Figure S8 — Changes in vaccination benefit for an increasing cattle-specific susceptibility and sheep-specific transmissibility. Smoothed image plot showing the changes in vaccination benefit (VB) in term of (A, C) infected premises and (B, D) epidemic duration when varying the cattle-specific susceptibility s cow and sheep-specific transmissibility t sheep. Simulations were generated considering a single infected premise at first detection and vaccinating cattle at day 7 as a complement to the culling of IP/DC premises. Figures show the evolution of the different measures for two counties: (A-B) Aberdeenshire and (C-D) Ayrshire as examples for the different dynamics identified in Scotland. The black circle shows the point at which scow/s sheep and tsheep/t cow take the Scotland-specific values fitted from the 2001 epidemic. (TIF) [file pone.0077616.s008.tif]

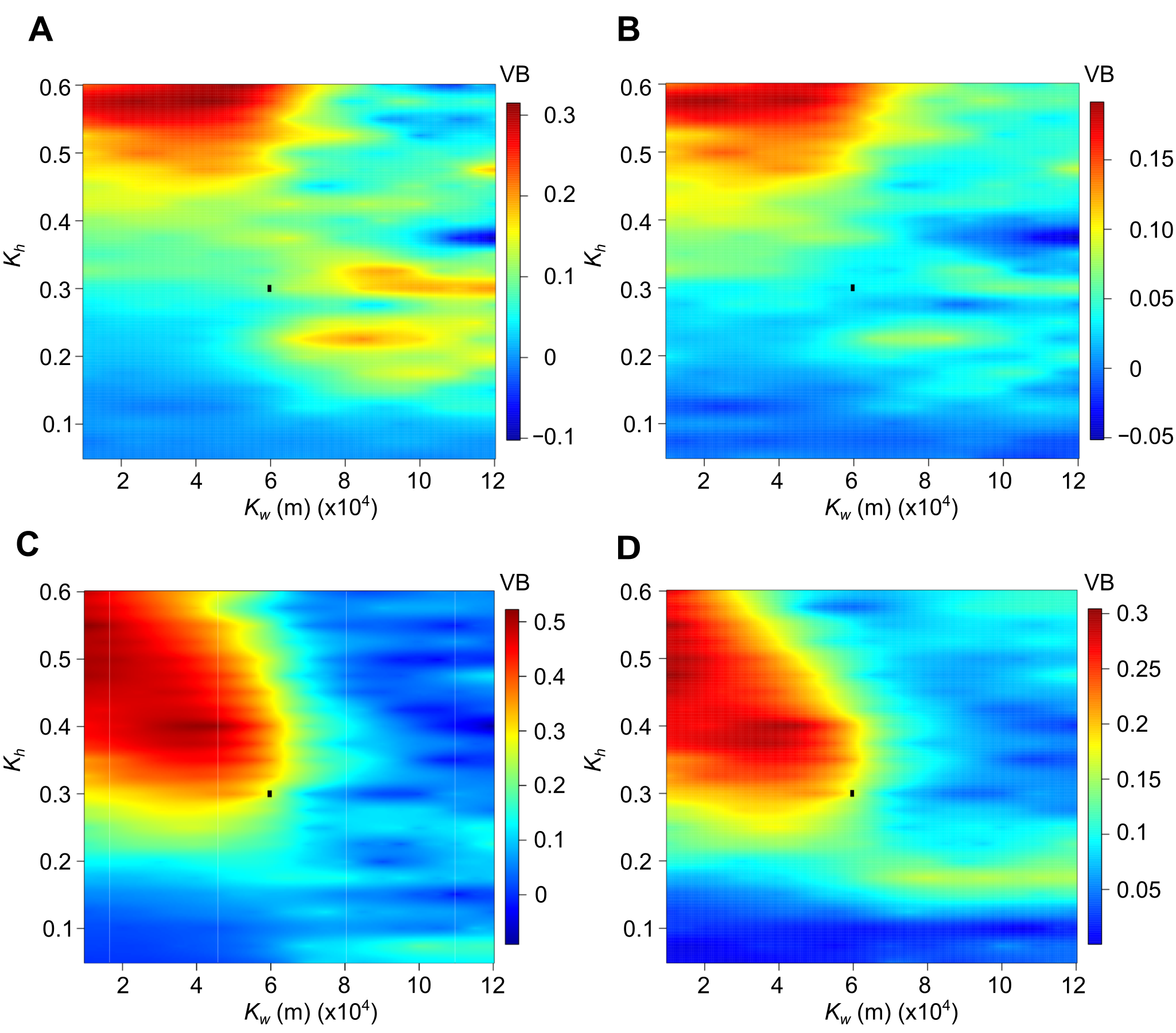

Supplement: Figure S9 — Changes in vaccination benefit (VB) when varying the transmission kernel shape. Smoothed image plot showing the changes in VB in term of infected premises (A, C) and epidemic duration (B, D) when varying the width (Kw) and height (Kh) of the transmission kernel function. Simulations were generated considering a single infected premise at first detection and vaccinating cattle at day 7 as a complement to the culling of IP/DC premises. Figures show evolution of the different measures for two counties: (A, B) Aberdeenshire and (C, D) Ayrshire as examples for the different dynamics identified in Scotland. The black circle shows the point at which K w and K h take the values observed during the 2001 epidemic. (TIF) [file pone.0077616.s009.tif]
