# Supplementary material for: Vaccination against Foot-And-Mouth Disease: Do Initial Conditions Affect Its Benefit?
Source: PLoS One. 2013 Oct 4;8(10):e77616. doi: 10.1371/journal.pone.0077616 (PMC3815046; doi:10.1371/journal.pone.0077616)
Supplement: Table S1 — Farm data for 2011 in Scotland and used in the model. (DOC) [file pone.0077616.s010.doc]

**Table S1. Farm data for 2011 in Scotland, as taken from the Scottish Agricultural Census June 2011 and as used in the model.** Note that the lower number of farms and animals that are considered in the model is due to the exclusion of Scottish islands.

|  | **Agricultural Census 2011** | **Model** |
| --- | --- | --- |
| **Cattle** |  |  |
| Number of farms | 12,455 | 10,934 |
| Median farm size (min-max) | 82 (1 - 6873) | 92 (1 - 6873) |
| Total number of animals (×1000) | 1803 | 1684 |
| **Sheep** |  |  |
| Number of farms | 14353 | 10113 |
| Median farm size (min-max) | 119 (1 - 14,278) | 195 (1 – 14,278) |
| Total number of animals (×1000) | 6785 | 6104 |
| **Pigs** |  |  |
| Number of farms | 1129 | 979 |
| Median farm size (min-max) | 4 (1 - 40373) | 4 (1 - 40373) |
| Total number of animals (×1000) | 383 | 382 |
| **Others (goats and deer)** |  |  |
| Number of farms | 646 | 571 |
| Median farm size (min-max) | 2 (1 - 486 ) | 2 (1 - 486) |
| Total number of animals | 8114 | 7824 |
